# Gut Microbiome Disruption in Traumatic Brain Injury and Therapeutic Potential of Probiotics: A Systematic Review and Meta-Analysis

**DOI:** 10.3390/biology15191730

**Published:** 2026-10-01

**Authors:** Zeeshan A. Khan, Zara Chowdhury, Riya Modi, Dewan Md. Sumsuzzman, Ling-Sha Ju, Rajendra K. Labala, Nikolaus Gravenstein, Anatoly E. Martynyuk

**Affiliations:** 1 Department of Anesthesiology, College of Medicine, University of Florida, Gainesville, FL 32610, USA; zkhan2@anest.ufl.edu (Z.A.K.); chowdhury.z@ufl.edu (Z.C.); riyamodi02@gmail.com (R.M.); lju@anest.ufl.edu (L.-S.J.); ngravenstein@anest.ufl.edu (N.G.); 2 Agent-Based Modelling Laboratory, Centre of Excellence in AI for Public Health Advancement, York University, Toronto, ON M3J 1P3, Canada; dewanp@yorku.ca; 3 Laboratory of Genomics and Bioinformatics, Institute of Molecular Genetics, Czech Academy of Sciences, 142 00 Prague, Czech Republic; rajendra-kumar.labala@img.cas.cz; 4 McKnight Brain Institute, College of Medicine, University of Florida, Gainesville, FL 32610, USA

**Keywords:** traumatic brain injury, probiotics, neurocognitive disorders, gut dysbiosis, inflammation

## Abstract

Background: Traumatic brain injury (TBI) is increasingly recognized as a systemic disorder that may involve alterations in the brain–gut-microbiome (BGM) axis, a bidirectional network linking the brain, immune system, and gut microbiota. While animal studies suggest that disruption and restoration of the BGM axis influence TBI-related changes and recovery, respectively, human evidence remains fragmented. Methods: MEDLINE, Embase, Scopus, and Web of Science were searched through 2025; Global Health was additionally searched for Aim 2. This study had two aims: (1) to assess gut microbiome changes following TBI and (2) to evaluate the effects of probiotic supplementation in TBI patients. Data were synthesized using random-effects meta-analyses, and risk of bias was assessed using the Newcastle–Ottawa and Cochrane tools. Results: Five studies (Aim 1) and fifteen studies (Aim 2) met the inclusion criteria. TBI was associated with shifts in microbial composition, including reduced abundance of *Prevotella* (*p* < 0.001), and *Parabacteroides merdae* (*p* = 0.038), whereas overall alpha diversity did not differ significantly. Probiotic supplementation improved neurological outcomes [Glasgow Coma Scale (GCS): MD = 2.25, 95% CI 1.34–3.16, *p* < 0.001] and functional outcomes [Glasgow Outcome Scale (GOS): MD = 1.40, 95% CI 1.12–1.69, *p* < 0.001], and reduced inflammatory markers, including TNF-α (*p* = 0.006), IL-6 (*p* < 0.001), procalcitonin (*p* < 0.001), and D-lactic acid levels (*p* < 0.001). High heterogeneity was observed across several outcomes, with the GOS showing moderate heterogeneity. Neurocognitive outcomes were evaluated qualitatively and were not pooled in a meta-analysis because of limited available data. Conclusions: The findings of this analysis suggest that TBI is associated with alterations in the gut microbiome and that microbiota-targeted therapies involving probiotics may improve inflammatory, gut-barrier, neurological, and functional outcomes. Although substantial heterogeneity was observed across several outcomes, particularly the GCS, the available evidence supports further investigation of microbiome-directed interventions as adjunctive strategies for TBI management. Standardized multicenter trials are warranted. PROSPERO: CRD420251135998.

## Simple Summary

Traumatic brain injury (TBI) is associated with changes in the gut microbiome, including reduced levels of specific beneficial bacteria. This review found that probiotic supplementation may reduce inflammation, enhance gut-barrier integrity, and improve neurological and functional recovery in patients with TBI. Overall, the findings suggest that targeting the gut microbiome could be a promising supportive strategy for TBI treatment, although larger, well-designed studies are needed to confirm these benefits.

## 1. Introduction

Traumatic brain injury (TBI) is a major global health concern and a leading cause of long-term disability, affecting millions of individuals each year [1]. Beyond the initial mechanical insult, TBI triggers a cascade of secondary injury processes, including neuroinflammation, oxidative stress, hypothalamic–pituitary–adrenal (HPA) axis dysregulation, and metabolic disturbances, that may underlie neurological and cognitive decline [2,3,4,5,6]. Increasingly, these secondary processes are understood to extend beyond the central nervous system, influencing systemic physiology through interconnected pathways such as the brain–gut-microbiome (BGM) axis [7,8].

The BGM axis plays a central role in maintaining physiological homeostasis through bidirectional communication involving neural, endocrine, immune, and metabolic signaling [9]. Disruptions of this axis have been implicated in a range of neuropsychiatric and neurodegenerative conditions, including depression, anxiety, autism spectrum disorder, and Alzheimer’s disease [10,11,12,13,14,15]. Within this framework, emerging evidence suggests that TBI may similarly disrupt gut microbial composition, diversity, and function, with downstream effects on systemic inflammation, intestinal barrier integrity, microbial metabolite production, and recovery trajectories [7,8].

Both primary studies and evidence syntheses support an association between brain injury and gut microbiome alterations. A recent systematic review [16] identified consistent reductions in microbial diversity and shifts in taxonomic composition across patients with acute brain injury, highlighting common microbiome responses to neurological insult. The strength of this work lies in its synthesis of converging signals across studies; however, the inclusion of heterogeneous injury populations and the absence of quantitative pooling limit the ability to draw TBI-specific and effect-size-based conclusions. Complementing these findings, meta-analyses of microbiome-targeted interventions provide preliminary evidence for clinical benefit. A recent study reported improvements in outcomes such as Glasgow Coma Scale (GCS) scores [17], while another demonstrated reductions in infection, mortality, gastrointestinal complications, and intensive care unit length of stay with probiotic-supplemented enteral nutrition [18]. These studies support the therapeutic potential of microbiome modulation, although variability in study design, probiotic formulations, dosing regimens, and outcome measures introduces heterogeneity that complicates direct comparison and generalizability.

Findings from original clinical studies further underscore both the promise and complexity of this field. Observational and interventional studies in TBI populations have reported alterations in microbial diversity and composition; however, the direction and magnitude of these changes are not fully consistent [19,20,21]. This variability likely reflects differences in injury severity, the timing of sample collection, sequencing methodologies, patient demographics, and comorbid conditions. Importantly, such heterogeneity highlights the sensitivity of the gut microbiome to both biological and methodological factors and underscores the need for standardized approaches to data collection and analysis.

Probiotic interventions have demonstrated immunomodulatory, barrier-protective, and cognitive benefits across a range of conditions [22,23,24,25,26]. In TBI, several clinical studies suggest potential benefits of probiotic supplementation; however, interpretation remains challenging due to small sample sizes, mixed patient populations (e.g., inclusion of polytrauma or non-TBI neurological injury), and variability in study quality and reporting [27,28,29,30,31,32]. While these studies provide important early signals, they also illustrate the need for a more rigorous and quantitatively integrated evaluation of outcomes, particularly those related to neurological and cognitive recovery. These considerations provide a clear rationale for a focused and methodologically robust synthesis of the evidence. Accordingly, the present systematic review and meta-analysis aims to: (1) evaluate alterations in gut microbiota composition and diversity in individuals with TBI; and (2) assess the therapeutic effects of probiotic supplementation on clinical outcomes in TBI populations.

## 2. Materials and Methods

### 2.1. Study Design and Registration

This systematic review and meta-analysis was conducted according to a predefined protocol registered in PROSPERO (CRD420251135998). The review has two analytical aims: (1) evaluation of alterations in gut microbiota composition and diversity associated with TBI and (2) assessment of the effects of probiotic supplementation on neurological outcomes (GCS), functional outcomes [Glasgow Outcome Scale (GOS)], and inflammatory biomarkers.

We conducted and reported this meta-analysis in accordance with the PRISMA guidelines for Systematic Reviews and Meta-Analyses and the synthesis without meta-analysis statements, respectively [33,34].

### 2.2. Data Sources and Search Strategy

A comprehensive literature search was conducted in Embase, MEDLINE, Scopus, and Web of Science, using Medical Subject Headings and free-text terms related to TBI, gut microbiota, dysbiosis, 16S rRNA gene sequencing, metagenomics, and probiotics. Additionally, the Global Health database was included in the Aim 2 search strategy. No restrictions on publication year, language, or study organism were applied to maximize the comprehensiveness and sensitivity of the search. Reference lists of all eligible studies were screened to ensure completeness. The full search strategy is provided in search strategy Supplementary File S1.

### 2.3. Inclusion and Exclusion Criteria

Studies were eligible under two predefined aims: for Aim 1, microbiome studies comparing diversity and gut microbial composition between individuals with TBI and healthy controls were included.

For Aim 2, studies evaluating the effects of probiotic supplementation on clinical outcomes in patients with TBI were included if they assessed neurological, functional, neurocognitive, gut-microbiome, and immunological outcomes. The participant pool comprised patients with a history of TBI receiving probiotic interventions and comparator groups not receiving probiotic supplementation (standard care controls).

Non-clinical or animal studies, reviews, and meta-analyses were excluded. For both aims, studies including all severities of TBI (mild, moderate, and severe) were considered eligible to avoid overlooking severity-dependent profiles of dysbiosis, should they exist. TBI severity was defined based on the GCS: mild (GCS 13–15), moderate (GCS 9–12), and severe (GCS ≤ 8) [35].

### 2.4. Study Selection

All records were screened and deduplicated using Rayyan [36], followed by structured manual verification based on predefined criteria (title, authors, year, DOI, abstract, and journal). Manual checks were applied to resolve ambiguous or incomplete records, ensuring accurate and reproducible duplicate removal. The deduplicated dataset was then screened using Rayyan based on titles and abstracts for relevance. Articles were independently reviewed by two investigators (Z.C. & R.M.), with disagreements resolved through consultation with a senior reviewer (Z.A.K.). All studies meeting inclusion criteria were archived in a secure shared drive at the University of Florida.

### 2.5. Data Extraction

For Aim 1, extracted variables included participant demographics, sequencing platform, sample size, means and standard deviations of alpha diversity metrics and relative abundance. Beta diversity was reported qualitatively using principal coordinate analysis (PCoA) plots with associated *p*-values. For Aim 2, extracted data included sample size, means and standard deviations, neurological and functional outcomes, probiotic strain(s), treatment duration, and inflammatory biomarkers. Graphical data were digitized using GetData Graph Digitizer [37].

A pilot extraction of 25% of studies was conducted to refine and standardize the extraction template, after which full data extraction was performed independently by two reviewers (Z.C. and R.M.), with discrepancies resolved through discussion or adjudication by a third reviewer (Z.A.K.) to ensure consistency and methodological rigor.

### 2.6. Quality Assessment

Observational studies were evaluated using the Newcastle–Ottawa Scale (NOS) [38], which evaluates three domains, selection (0–4 stars), comparability (0–2 stars), and outcome (0–3 stars), yielding a total score of 0–9 stars; studies scoring ≥ 7 were considered high-quality, 5–6 moderate-quality, and ≤4 low-quality. Randomized, controlled trials (RCTs) were evaluated using the Cochrane Risk of Bias 2 (RoB 2) tool, assessing five domains: bias arising from the randomization process, deviations from intended interventions, missing outcome data, measurement of the outcome, and selection of the reported result; studies were judged as low risk, some concerns, or high risk of bias, with assessments conducted independently by two reviewers and discrepancies resolved through discussion or third-reviewer adjudication.

### 2.7. Statistical Analysis

Quantitative syntheses were conducted in R (4.6.0) using the meta package. For microbiome outcomes, standardized mean differences (SMDs) were calculated for alpha diversity metrics and for relative abundance of taxa.

All relative abundance values were first harmonized to the proportion scale (0–1); studies reporting percentages were divided by 100, with the same conversion applied to their standard errors. Because the included studies reported only group-level summary statistics (mean relative abundance and standard error of the mean) rather than individual sample-level abundance profiles, compositional transformations such as the centered log-ratio could not be applied, as these require the full per-sample taxon abundance vector.

To maximize comparability across studies reporting different taxonomic resolutions, related sub-taxa were aggregated to common phylum, family, and genus levels. Abundances were summed and standard error means (SEMs) were propagated using the root-sum-of-squares approach (SEM_combined = √(Σ SEM_i^2^), following standard error-propagation principles for sums under the assumption of independence among the component estimates. Because covariance information between sub-taxa was not reported in the original publications, direct calculation of the variance of the summed abundance was not feasible. The propagated SEM therefore represents an approximation that is expected to be conservative when covariance is negligible. Standard errors were then converted to standard deviations (SD = SEM × √*n*) using each study’s reported group sample sizes. Standardized mean differences (Hedges’ g) were computed for each taxon individually using the metacont function in the R meta package, which divides the between-group mean difference by the pooled standard deviation and applies a small-sample correction. Random-effects models with restricted maximum likelihood estimation were used throughout. All beta diversity results were reported using PCoA and associated *p*-values, which did not allow us to perform a meta-analysis. For probiotic treatment outcomes, mean differences (MDs) were calculated for neurological outcomes (GCS), functional outcomes (GOS) and inflammatory biomarkers. All analyses employed random-effects models with restricted maximum likelihood estimation.

The predictive interval (PI) was calculated using the following equation:

PI = µ ± t_df,1−α/2_ × √τ^2^ + SE(μ)^2^

where µ = pooled effect estimate;

τ^2^ = between-study variance;

SE(μ) = standard error of the pooled estimate;

df = degrees of freedom;

α = significance level (0.05 for a 95% PI).

Statistical heterogeneity was quantified using the I^2^ and τ^2^ statistics, with I^2^ thresholds interpreted as low (25%), moderate (50%), and high (75%) heterogeneity, while τ^2^ provided an estimate of between-study variance in effect sizes. Robustness of pooled estimates was assessed through leave-one-out sensitivity analyses. A separate risk-of-bias sensitivity analysis excluded studies rated high or some/high risk of bias. Meta-regression was conducted to examine whether probiotic treatment duration or publication year explained variability in GCS outcomes. Potential publication bias was explored using funnel plots; formal tests of funnel plot asymmetry were interpreted cautiously when fewer than 10 studies were available. For all outcomes, quantitative syntheses were conducted separately within the same study design, such that randomized, controlled trials were pooled only with randomized, controlled trials and observational studies only with observational studies. The certainty of the evidence for the GCS and GOS was assessed using the GRADE approach.

## 3. Results

### 3.1. Study Search and Selection

For Aim 1, the literature search identified a total of 1212 records, of which 317 duplicates were removed prior to screening. Of the 895 unique records screened, 858 were excluded at the title/abstract stage, and 37 full-text articles were sought for retrieval. Data from two full-text articles could not be obtained despite follow-up attempts and email inquiries, leaving 35 studies assessed for eligibility. Following detailed evaluations, 30 studies were excluded for reasons including an irrelevant population such as non-traumatic brain injury (*n* = 15), an irrelevant article type such as a review article (*n* = 6), irrelevant outcomes not reporting microbiome changes (*n* = 5) and incomplete trials (*n* = 4) (Table S1A). Ultimately, five studies met the inclusion criteria and were included in the meta-analysis [19,20,21,39,40] (Figure 1).

For Aim 2, the literature search returned 7105 records, of which 2522 duplicates were removed prior to screening. A total of 4583 unique records were screened by title and abstract, resulting in the exclusion of 4538 records that did not meet the basic inclusion criteria. One additional record was identified through systematic reference checking. In total, 46 full text-articles were sought for retrieval, and all were successfully obtained. These 46 articles were assessed for eligibility, and 31 studies were subsequently excluded for the following reasons: irrelevant interventions, such as enteral nutrition or nutrient-rich diets only (*n* = 8); irrelevant populations, including studies without a control, patients with cerebral hemorrhage or those in the acute stress stage following neurosurgery or intracranial trauma (*n* = 5); inappropriate study designs, such as case reports (*n* = 7); irrelevant publication types, including review articles (*n* = 4); irrelevant outcomes, such as change in body weight, glucose level, and biomarkers of cellular integrity (*n* = 5); an animal study (*n* = 1); and a retracted article (*n* = 1) (Table S1B). Ultimately, 15 studies met the inclusion criteria and were incorporated into the qualitative synthesis and meta-analysis [28,29,31,41,42,43,44,45,46,47,48,49,50,51,52].

### 3.2. Study Characteristics

For Aim 1, five studies published between 2020 and 2024 were included in Aim 1 [19,20,21,39,40] (Table 1A). All studies were conducted in the United States, reflecting the geographic concentration of microbiome-focused TBI research during this period. Cohorts were predominantly male across all five studies, consistent with the well-established male predominance in TBI epidemiology [53,54]. Mean participant ages ranged from 19.3 to 52.7 years. BMI was reported in four of the five studies [20,21,39,40]. Mean BMI values ranged from 28.2 ± 4.1 to 33.0 ± 4.7 kg/m^2^, spanning both the overweight and obesity categories; two of the four cohorts reported mean BMI values ≥ 30 kg/m^2^.

TBI severity profiles differed across studies. Three studies focused on individuals with moderate-to-severe TBI [20,21,39], one examined mild-to-moderate TBI [40], and one enrolled participants across mixed severity categories spanning mild, moderate, and severe injury [19] (Table 1A).

Microbiome assessment methods varied across studies. Three studies employed 16S rRNA gene sequencing [21,39,40], targeting either the V4 hypervariable region or the broader V1-V9 region, to characterize alpha diversity, beta diversity, and taxonomic composition. One study used a qPCR array to evaluate metagenomic stability and metatranscriptomics to characterize functional microbial profiles [20]. One study applied quantitative PCR-based approaches to quantify specific microbial taxa [19] (Table 1A).

For Aim 2, fifteen studies published between 2007 and 2024 were included [28,29,31,41,42,43,44,45,46,47,48,49,50,51,52]. The majority of studies were conducted in China, with one study each from Iran [41] and the United States [49]. Notably, none of the thirteen included studies from China performed gut microbiome sequencing, limiting direct characterization of probiotic-associated compositional changes. Most studies enrolled adults with severe TBI, typically defined as a GCS score ≤ 8, as this population presents the most pronounced physiological disruptions, including systemic inflammation, immune dysregulation, and gut barrier compromise, that represent mechanistically plausible targets for probiotic intervention. The sole exception was a United States-based study [49], which examined patients with mild TBI. Participant ages were broadly comparable across treatment and control groups, with mean ages in probiotic groups ranging from approximately 33.5 to 49.7 years and in control groups from 31.3 to 50.3 years. A male predominance was observed across studies, consistent with TBI epidemiology. BMI was infrequently reported, available in only three studies [29,41,48], with mean values of approximately 22–25 kg/m^2^.

All included studies compared a probiotic intervention group against a control group receiving standard care [28,29,31,41,42,43,44,45,46,47,48,49,50,51,52]. Outcome measures were broad and multimodal. Neurological recovery was assessed using the GCS [29,41,42,43,45,46,47,52], and functional recovery through the GOS was reported in [31,44,50]; the pooled GOS analysis included the observational studies [44,50]. The Mini-Mental State Examination and the Montreal Cognitive Assessment were also conducted [28]. Immune outcomes were evaluated primarily through flow cytometry, characterizing Th17/Treg [41], and CD3+/CD4+ T-cell profiles [51]. Inflammatory burden was assessed via ELISA, measuring cytokines including Interleukin-6 (IL-6) [29,43,44,45,48], Tumor Necrosis Factor-alpha (TNF-α) [29,41,43,44], IL-10 [41,48], C-reactive protein (CRP) [47,48], circulating CD3+ and CD4+ T-cell [29]. Gut-barrier integrity was evaluated through serum D-lactic acid [28,29,44,50]. The procalcitonin level was measured to assess systemic bacterial infection [28,29,44,50]. Immune competence was further assessed via serum IL-8 [29,45] and IgA, IgG, and IgM [42,51], although these outcomes were reported in only one RCT and/or one observational study. One study employed 16S rRNA gene sequencing to assess probiotic-associated shifts in microbiome composition [49]. Full study details are summarized in Table 1B.

### 3.3. Risk of Bias and Quality Reporting

For Aim 1, all included studies demonstrated a low risk of bias, each receiving a minimum score of seven out of nine stars. Two studies received seven of nine stars [20,21] and three studies received eight of nine stars [19,39,40]. Accordingly, all studies were classified as high-quality, indicating a low overall risk of bias (Table 2).

Table 2 shows the Newcastle–Ottawa Scale quality assessment that was used to evaluate the risk of bias for each included study in Aim 1. Under each criterion, an article was awarded an ‘*’ if it met the criteria, or a ‘/’ if it fell short of the criterion for that category. Scoring was based on the number of ‘*’s out of a possible nine. A higher number of ‘*’s indicated a lower risk of bias.

For Aim 2, eleven studies were RCTs [29,31,41,42,43,45,46,47,48,49,52], and four were observational studies [28,44,50,51]. The observational studies were assessed using the NOS, and all four demonstrated a low risk of bias, each receiving a minimum score of seven out of nine stars. Among the RCTs, three [41,47,48] were judged to be at low risk of bias. Five of the studied RCTs [29,43,46,49,52] were judged to have some concerns. Studies [31,45] were rated as some-to-high-risk, and [42] was classified as a high-risk study (Table 3A).

Table 3 shows the risk of bias (RoB) analysis for Aim 2. (A) The Cochrane RoB version 2 tool was used to assess the quality of randomized control trials (RCTs). Studies were categorized as having low, some, or high risk of bias. (B) Newcastle–Ottawa Scale quality assessment was used to evaluate the risk of bias for each included study in Aim 2. Under each criterion, an article was awarded an ‘*’ if it met the criteria, or a ‘/’ if it fell short of the criterion for that category. Scoring was based on the number of ‘*’s out of a possible nine. A higher number of ‘*’s indicated a lower risk of bias.

### 3.4. Altered Shannon Diversity Revealed Through Alpha Diversity Sensitivity Analysis

Richness assessment using observed Operational Taxonomic Units (OTUs) [21,39] showed a non-significant SMD 0.04 (95% CI −0.63–0.72; *p* = 0.905, I^2^ = 69.6%, Figure 2A). Richness–evenness indices using Shannon diversity [21,39,40] also yielded a non-significant SMD 0.64 (95% CI −0.40–1.68; *p* = 0.230, I^2^ = 90.2%, Figure 2B). Interestingly, leave-one-out analysis revealed a significant alteration in richness–evenness with low heterogeneity (SMD 1.13; 95% CI 0.54–1.73; *p* < 0.001, I^2^ = 30.0%) (Figure S1).

### 3.5. Depletion of Prevotella and Parabacteroides merdae Revealed Through Taxonomic Meta-Analysis

Analysis of relative abundance of specific gut microbial taxa revealed statistically significant differences across genus and species levels. At the genus level, *Prevotella* was also significantly reduced following TBI, showing a pooled SMD of −0.87 (95% CI −1.33 to −0.40, *p* < 0.001, I^2^ = 0.0%; Figure 3A). The consistency of effect estimates across studies further supports a robust association between TBI and depletion of *Prevotella*-related taxa.

At the species level, *Parabacteroides merdae* demonstrated significant depletion in the TBI group, with a pooled SMD of −0.67 (95% CI −1.30 to −0.04, *p* = 0.038, I^2^ = 6.5%; Figure 3B). Heterogeneity was low, indicating good agreement between the included studies. Collectively, these findings suggest that TBI is associated with a reduction in specific members of the gut microbiota, particularly taxa within the *Prevotella* lineage and *Parabacteroides merdae*. Although the effect sizes were moderate, the consistent direction of change across studies supports the biological relevance of these alterations in the post-TBI gut microbial community.

### 3.6. Incompatible Data Structures Prevent Beta-Diversity Meta-Analysis

The absence of extractable numerical data, including effect size estimates and compatible distance matrices, precluded beta-diversity meta-analysis. Briefly, one study [21] observed changes in both unweighted and weighted UniFrac distances, while another [40] reported differences in weighted UniFrac between control and TBI patients. In contrast, [39] found no significant differences in any beta diversity metrics, including Bray–Curtis, unweighted UniFrac, and weighted UniFrac.

### 3.7. Improved Neurological and Functional Outcomes Revealed Through Probiotic Supplementation Meta-Analysis

Evaluation of neurological outcomes showed that probiotic supplementation was associated with significant improvements in the GCS [29,41,42,43,45,46,47,52]. As only one study included patients with mild TBI and no studies included patients with moderate TBI, the meta-analysis was restricted to studies enrolling patients with severe TBI. Restricting the analysis to severe TBI may have reduced clinical heterogeneity related to injury severity by focusing on a population characterized by pronounced systemic inflammation, immune dysregulation, and gut-barrier dysfunction, which are key mechanistic targets of probiotic intervention. Nevertheless, substantial clinical and methodological heterogeneity remained across studies. Across eight studies (*n* = 782), the pooled mean difference favored probiotics with an improvement of 2.25 points (95% CI 1.34–3.16, *p* < 0.001, I^2^ = 97.3%, Figure 4A). Between-study heterogeneity remained very high (I^2^ = 97.3%). Moreover, the 95% prediction interval (−0.88 to 5.38) crossed the null, indicating substantial uncertainty in the magnitude and direction of the effect that might be observed in a future comparable study. Meta-regression analyses using intervention duration and publication year as moderators showed no statistically significant associations with GCS outcomes. Intervention duration (10–28 days) was not significantly associated with the magnitude of the GCS treatment effect (β = −0.010, 95% CI −0.193 to 0.172; *p* = 0.911) and explained none of the observed between-study heterogeneity (R^2^ = 0%). Likewise, publication year (2007–2024) was not significantly associated with GCS outcomes (β = −0.037, 95% CI −0.228 to 0.153; *p* = 0.701) and similarly explained none of the observed heterogeneity (R^2^ = 0%) (Figure 4B,C).

Furthermore, sensitivity analysis across six studies (*n* = 576) after excluding studies rated high or some/high risk of bias did not materially reduce heterogeneity, and the overall effect remained statistically significant (MD = 2.04, 95% CI 0.93 to 3.16; *p* < 0.001; I^2^ = 94.9%) (Figure S2). These findings suggest that the direction of the pooled effect was not materially altered after exclusion of higher-risk studies; however, the persistently high heterogeneity lowers confidence in the precision and generalizability of the pooled effect estimate.

Consistent with the GCS findings, analysis of the GOS [44,50] across two studies (*n* = 213) demonstrated a pooled mean difference favoring probiotic treatment, with an improvement of 1.40 points (95% CI 1.12–1.69, *p* < 0.001, I^2^ = 40.7%), with moderate heterogeneity (Figure 4D).

### 3.8. Modulated Systemic Inflammation and Gut-Barrier Function Revealed Through Probiotic Supplementation Analysis

Inflammatory and gut-barrier outcomes following probiotic intervention revealed biologically coherent effects favoring treatment, but with high, unexplained heterogeneity. Probiotics were associated with significant reductions in TNF-α [29,41,43] −1.81 (95% CI −3.09–−0.52, *p* = 0.006, I^2^ = 93.7%, Figure 5A), IL-6 [29,43,48] −20.11 (95% CI −31.57–−8.66, *p* < 0.001, I^2^ = 72.7%, Figure 5B), and procalcitonin [28,44,50] −2.40 (95% CI −3.03–−1.78, *p* < 0.001, I^2^ = 93.4%, Figure 5C), indicating attenuation of systemic inflammation and infection-related responses, but with high heterogeneity. Similarly, serum D-lactic acid [28,44,50], a marker of impaired gut-barrier integrity, showed a marked downregulation in probiotic-treated patients (MD = −0.98, 95% CI −1.19–−0.76, *p* < 0.001, I^2^ = 92.1%) (Figure 5D). In contrast, other inflammatory and immune parameters, such as IL-10 (Figure S3A), CRP (Figure S3B), and circulating CD3+ (Figure S3C) and CD4+ T-cell counts (Figure S3D), showed no significant differences between probiotic and control groups.

### 3.9. Publication Bias

An exploratory funnel-plot assessment and Egger’s regression test using the eight GCS studies [29,41,42,43,45,46,47,52] showed no statistically significant evidence of small-study effects (Egger intercept *p* = 0.087) (Figure S4); interpretation is limited by the small number of studies.

### 3.10. Certainty of the Evidence

The certainty of the evidence for the efficacy of probiotic intervention on the GCS and GOS was assessed using the GRADE approach. In the current domain-level GRADE assessment, both the GCS and GOS were rated as low-certainty (Table S5A,B).

### 3.11. Systematic Review of Neurocognitive Recovery Following Probiotic Intervention

One study [28] reported significant cognitive improvements using objective screening tools in TBI patients with probiotic supplementation. On the Mini-Mental State Examination, probiotic-treated patients achieved scores of 24.65 ± 2.08 compared with 22.16 ± 1.95 in controls (*p* < 0.001). Similarly, Montreal Cognitive Assessment (MoCA) scores were higher in the probiotic group (26.61 ± 2.17) than in controls (24.35 ± 1.83, *p* < 0.001) [28]. These findings indicate statistically and clinically meaningful improvements in global cognition associated with probiotic supplementation (Table S6). Another study [49] reported only baseline Neurobehavioral Symptom Inventory scores and did not provide any post-intervention analysis.

This systematic review suggests that probiotic supplementation may have beneficial effects on neurocognitive function recovery. Objective cognitive assessments demonstrated positive outcomes following probiotic intervention; however, the evidence is limited, as only one study with a small sample size was identified, which precludes the generalizability of these findings. Therefore, further well-designed randomized, controlled trials employing standardized neuropsychological assessment batteries are needed to confirm the efficacy of probiotic supplementation in enhancing neurocognitive recovery.

## 4. Discussion

Our meta-analysis identified reproducible alterations in the gut microbiome of adults with TBI. While the primary analysis showed no significant differences in pooled alpha diversity indices between TBI patients and controls, the omission of study [39] in sensitivity analyses resulted in a significant increase in Shannon diversity and markedly reduced heterogeneity [21,40]. The excluded study evaluated military veterans [39], whereas the other studies included civilian populations [21,40]. The differences in population characteristics may have contributed to both the observed heterogeneity and attenuation of the pooled effect estimate. The emergence of a statistically significant effect following exclusion of this study suggests that TBI-associated alterations in microbial community structure may be present; however, given that only three datasets were available and the significance depended on removal of a single study, these findings should be interpreted with caution. Overall, the results indicate that while species richness may be largely preserved, microbial evenness and ecological organization may be disrupted following TBI.

Meta-analysis of taxonomic relative abundance further demonstrated significant depletion of the genus *Prevotella* [20,21,40] and species *Parabacteroides merdae* [20,21] in TBI patients, taxa commonly associated with gut homeostasis. *Prevotella* and *Parabacteroides merdae* have been implicated in epithelial barrier maintenance, carbohydrate fermentation, anti-inflammatory signaling, and short-chain fatty acid production in the prior literature; however, their functional consequences were not directly assessed in the included studies. The observed depletion of these taxa may therefore reflect a microbiome configuration associated with impaired mucosal resilience and pro-inflammatory signaling [55,56]. Given the established role of neuroinflammation in secondary brain injury [57,58], gut dysbiosis may represent a contributory, yet incompletely defined, factor in post-TBI inflammatory cascades.

Altered microbial community structure and depletion of taxa commonly associated with gut homeostasis may contribute to dysregulation of the stress–immune axis, altered HPA axis signaling, and impaired recovery following TBI; however, these mechanisms were not directly assessed in the included studies and therefore remain hypothetical [59,60,61]. The emerging literature linking gut dysbiosis to neurodegenerative and cognitive vulnerability raises the possibility that these microbial signatures could serve as early indicators of long-term neurological risk in TBI survivors, although this concept requires prospective validation [15,62,63,64,65,66].

Interpretation of these findings must be tempered by important methodological limitations, including the small number of available studies, limited geographic representation, and substantial heterogeneity in sequencing platforms, analytical pipelines, and outcome measures. Standardized microbiome methodologies and multicenter longitudinal cohorts will be necessary to validate these microbial patterns and clarify their temporal evolution after injury. In addition, the Soriano et al. [40] alpha diversity dataset included longitudinal samples from only four concussed participants; repeated observations may therefore not be fully independent, and the corresponding pooled estimates should be interpreted cautiously.

In agreement with the hypothesis that TBI is associated with gut microbial disruption, the interventional component of this meta-analysis revealed that probiotic supplementation was associated with improvements in neurological outcomes and favorable modulation of systemic inflammatory markers. Notably, the pooled improvement of 2.25 points in the GCS may represent a clinically meaningful improvement in consciousness level, particularly in patients with moderate-to-severe impairment. Even modest gains in the GCS may facilitate neurological assessment and monitoring during critical illness; however, the very high heterogeneity observed across studies (I^2^ = 97.3%) warrants cautious interpretation of these findings. Even though the observed benefits of probiotics may be mediated through alterations in the gut microbiome, direct evidence remains limited because most included studies did not assess microbiome changes following probiotic treatment. Therefore, post-probiotic microbiota profiling is required to confirm this hypothesis.

Differences in baseline characteristics, probiotic strains (single- vs. multi-strain formulations), colony-forming unit dosing, the timing of intervention initiation, and outcome measurement mostly contribute to the observed variability across studies.

Importantly, meta-regression did not identify intervention duration as a significant source of heterogeneity, indicating that treatment length alone does not account for differences in effect magnitude. Instead, factors such as strain specificity, baseline host microbiome composition, antibiotic exposure, and nutritional protocols may play more substantial roles. In particular, antibiotics may directly alter microbiome diversity independently of TBI. Antibiotic exposure was inconsistently reported across studies and therefore could not be systematically examined. Future trials would benefit from standardized strain selection, clearly defined dosing regimens, and harmonized neurological assessment time points.

In contrast, functional recovery assessed by the GOS demonstrated improvement with moderate heterogeneity (I^2^ = 40.7%), thereby increasing confidence in the consistency of this finding across studies. The GOS is a global functional measure that categorizes outcomes from death to good recovery and incorporates elements of independence, social reintegration, and neurobehavioral function. Unlike the GCS, which reflects momentary levels of consciousness and is influenced by sedation and acute physiological factors, the GOS captures broader functional status and longer-term recovery.

The moderate heterogeneity observed in GOS outcomes suggests that the association between probiotic supplementation and global functional recovery was relatively consistent despite differences in study design, probiotic formulation, and patient characteristics. This consistency reduces concern that the observed effect is driven by outlier studies. While the underlying mechanisms cannot be determined from the available data, these findings raise the possibility that broader functional recovery may be less sensitive to short-term clinical fluctuations and more closely associated with systemic physiological processes, such as inflammation and metabolic regulation, that are known to be influenced by gut microbiome modulation.

Improvements in the GOS are clinically meaningful, as even a one-category shift (e.g., from severe disability to moderate disability) reflects substantial gains in functional independence, reduced caregiver burden, and improved quality of life. The moderate heterogeneity suggests greater consistency than observed for the GCS, although confidence remains limited by the small observational evidence base.

Although both the GCS and GOS improved following probiotic intervention, confidence in these findings remained limited. The current GRADE assessment rated both outcomes as low-certainty evidence (⊕⊕○○), although for different reasons. Certainty for the GCS was downgraded because of substantial heterogeneity and imprecision, whereas the GOS evidence was based on only two observational studies (*n* = 213), limiting the breadth and generalizability of the evidence despite a relatively consistent pooled effect and a confidence interval that excluded the null. Accordingly, the observed improvement in the GOS should be interpreted cautiously. Additional well-designed prospective studies and randomized, controlled trials are needed to strengthen confidence in the effect of probiotic supplementation on functional recovery across diverse TBI populations.

Biomarker analyses offer biologically plausible context for these clinical findings. Probiotic supplementation was associated with reductions in pro-inflammatory mediators such as TNF-α [29,41,43] and procalcitonin [28,44,50], suggesting attenuation of systemic inflammatory activity. In contrast, pooled analyses of IL-10, CRP, CD3+, and CD4+ did not show statistically significant effects. Nevertheless, these findings should be interpreted with caution, as substantial heterogeneity was observed across studies, limiting confidence in the pooled estimates and making it difficult to exclude potentially meaningful effects. Furthermore, a decrease in serum D-lactic acid levels [28,44,50], a marker of impaired intestinal barrier integrity, may indicate improved gut barrier function and reduced intestinal permeability following probiotic supplementation. Although this finding supports the barrier-protective effects of probiotics, the high heterogeneity observed across studies limits its generalizability.

Taken together, the observational and interventional findings suggest that TBI is associated with gut microbiome alterations characterized by changes in community structure and depletion of taxa commonly linked to gut homeostasis, and that probiotic supplementation is associated with improvements in selected inflammatory, gut-barrier integrity, and neurological outcomes. These findings are consistent with a bidirectional BGM framework in which neurotrauma may disrupt intestinal ecology, with potential downstream implications for systemic inflammation and in neurological recovery; however, direct causal relationships cannot be established from the available data.

The taxa identified as depleted, particularly *Prevotella* and *Parabacteroides merdae*, represent biologically plausible contributors to gut homeostasis based on the prior literature, but their role as therapeutic targets remains speculative. Rationally designed probiotic or prebiotic strategies aimed at modulating these microbial communities may hold promise for enhancing metabolic resilience and reducing inflammatory burden, although heterogeneity across trials and limited sample sizes indicate that routine clinical implementation is premature despite a favorable risk/cost benefit profile of probiotic supplementation.

Future research should integrate microbiome profiling with interventional trial designs to identify responder phenotypes, evaluate strain-specific efficacy, and clarify underlying causal pathways. Large, multicenter RCTs employing standardized microbiome methodologies and harmonized clinical outcome measures will be essential to translate these preliminary findings into evidence-based therapeutic approaches.

Consistent with other systematic reviews in this field, the PRISMA flow diagram demonstrates that although the initial literature search yielded a large number of records, only a small subset met the methodological and conceptual criteria required for rigorous evaluation. This underscores both the limited high-quality evidence base and the need for cautious interpretation when synthesizing findings related to probiotic or microbiome-targeted interventions in TBI.

## 5. Conclusions

In conclusion, this meta-analysis suggests that TBI is associated with biologically relevant alterations in the gut microbiome. Moreover, the available evidence indicates that probiotic supplementation may contribute to improved neurological recovery in patients with TBI. The observed improvements in the GOS were modest but may be clinically meaningful. Although substantial heterogeneity limits definitive conclusions, the convergence of microbial, inflammatory, and clinical findings highlights the potential of microbiome-directed strategies as adjunctive approaches in TBI rehabilitation.

## Figures and Tables

**Figure 1:**
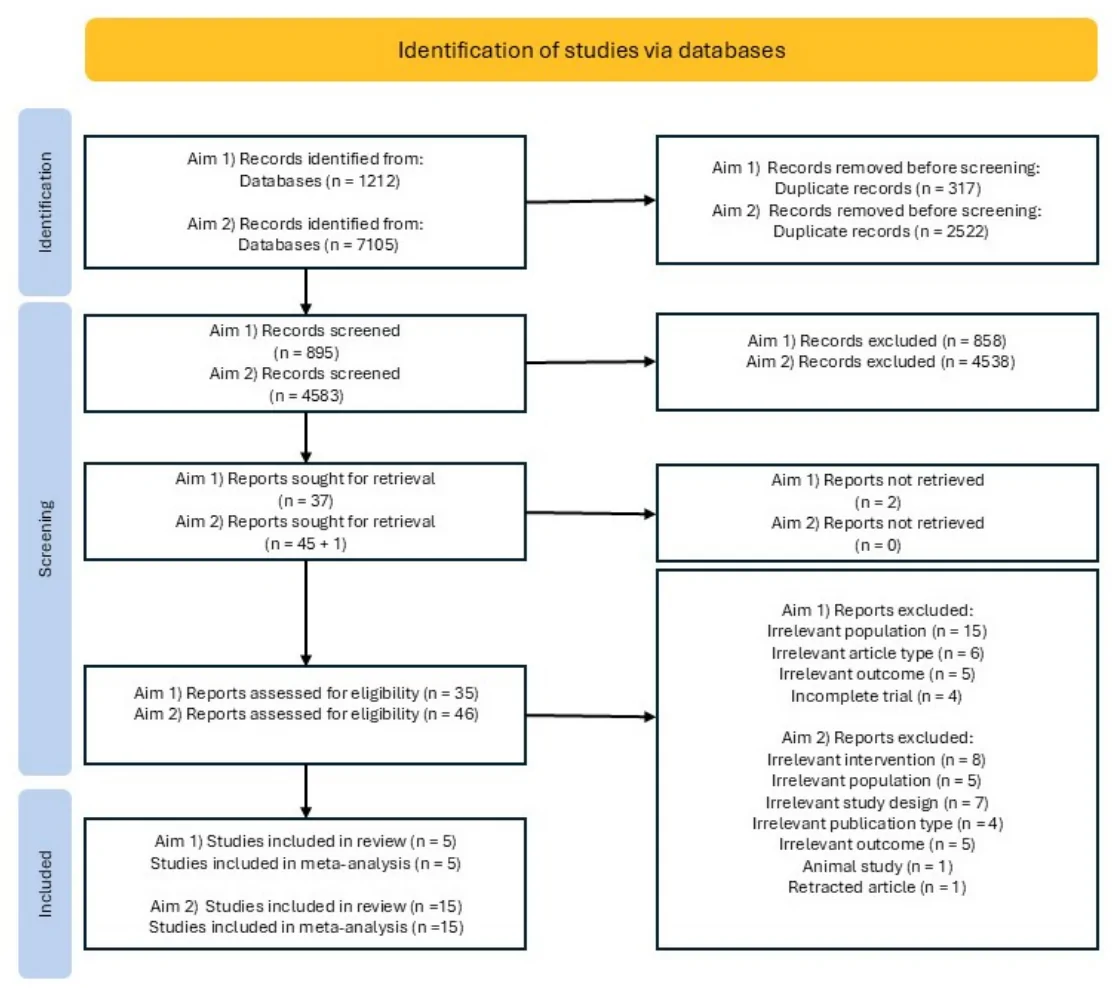
This diagram outlines the screening and selection procedures for identifying eligible studies. For Aim 1, a total of 1212 records were identified from various databases. After removing 317 duplicates, 895 records underwent title/abstract screening, with 858 excluded for irrelevance. Thirty-seven full text reports were assessed; two were not retrievable. Of the remaining 35, 30 were excluded due to irrelevant population (*n* = 15), irrelevant article type (*n* = 6), irrelevant outcomes (*n* = 5), or incomplete trial (*n* = 4). Five observational studies met all eligibility criteria and were included in this review. For Aim 2, a total of 7105 records were identified from various databases. After removing 2522 duplicates, a total of 4583 unique records were screened by title and abstract, resulting in the exclusion of 4538 records that did not meet basic inclusion criteria. One additional record was identified through systematic reference checking. In total, 46 full-text articles were sought for retrieval, and all were successfully obtained. These 46 articles were assessed for eligibility, with 31 studies excluded for the following reasons: irrelevant intervention (*n* = 8), irrelevant population (*n* = 5), irrelevant study design (*n* = 7), irrelevant publication type (*n* = 4), irrelevant outcomes (*n* = 5), animal study (*n* = 1), and retracted article (*n* = 1). Finally, 15 studies were selected for Aim 2.

**Figure 2:**
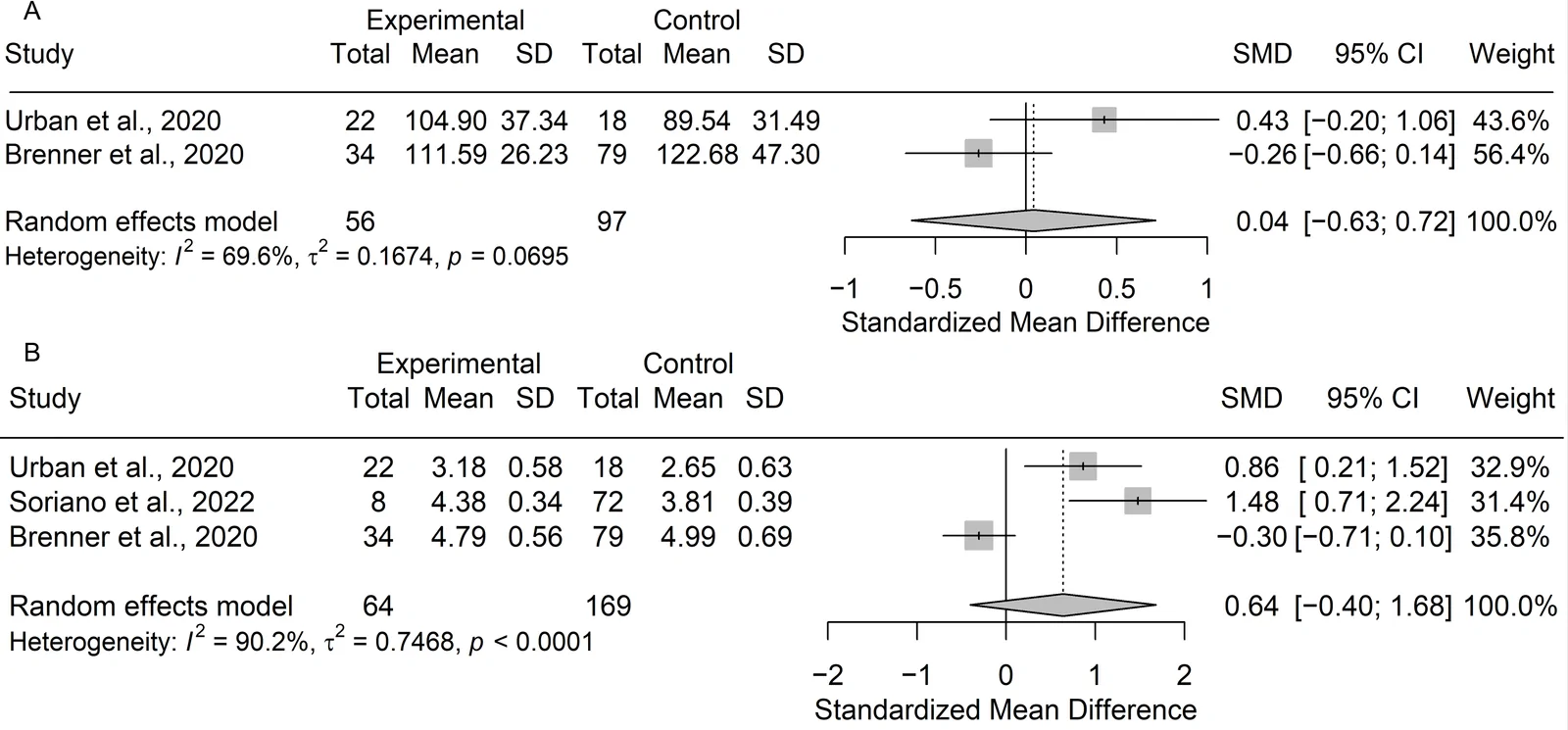
This figure summarizes standardized mean differences for several alpha diversity indices evaluated in TBI-related microbiome analyses. (**A**) Richness metrics from two studies. The pooled estimate was small and non-significant, accompanied by moderate heterogeneity. (**B**) Shannon diversity (richness/evenness) from three datasets [21,39,40]. The pooled effect suggested a moderate but non-significant increase in Shannon diversity. Squares represent individual study effect sizes (SMD) with 95% confidence intervals (CIs), with marker size proportional to study weight; horizontal lines indicate 95% CIs. Diamonds denote pooled estimates from random-effects models, with widths representing 95% CIs. Heterogeneity is reported by I^2^ and corresponding *p*-values.

**Figure 3:**
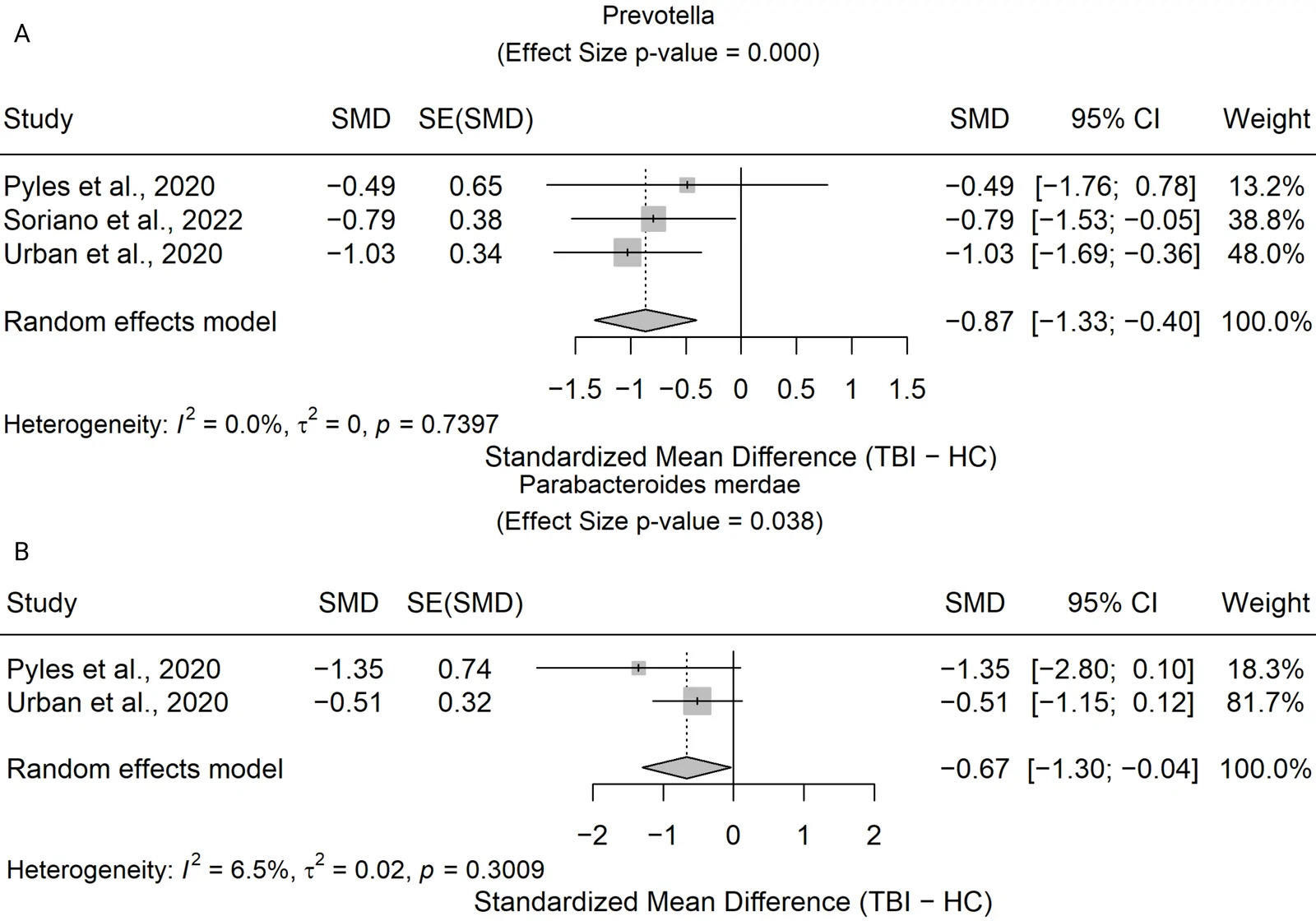
(**A**) Relative abundance of the genus *Prevotella* across three studies showed a significantly lower pooled estimate in TBI compared with control groups, with low, non-significant heterogeneity. (**B**) Relative abundance of the species *Parabacteroides merdae* across two studies showed a significantly lower pooled estimate in TBI compared with control groups, with low, non-significant heterogeneity [20,21,40]. Squares represent individual study effect sizes (SMD) with 95% confidence intervals (CIs), with marker size proportional to study weight; horizontal lines indicate 95% CIs. Diamonds represent pooled estimates generated using random-effects models, with widths corresponding to 95% CIs. Statistical heterogeneity is quantified using I^2^ with associated *p*-values. No significant differences in relative abundance were detected across multiple phyla, genera and species (Tables S2–S4).

**Figure 4:**
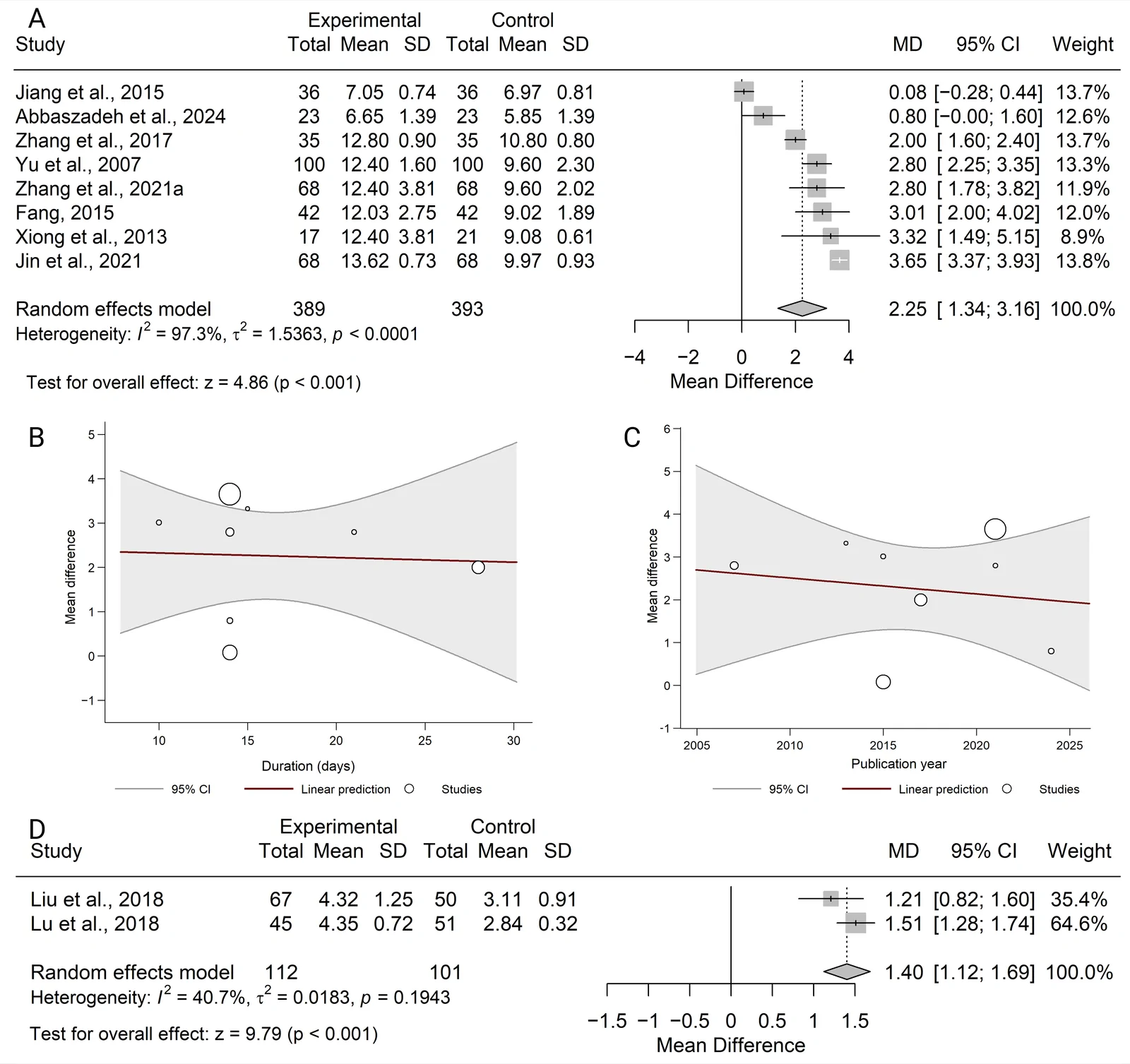
(**A**) Glasgow Coma Scale (GCS) Forest Plot: Eight studies (n = 782) comparing probiotics versus standard nutrition are shown. The pooled mean difference favored probiotics (MD = 2.25, 95% CI 1.34–3.16, *p* < 0.001), indicating improvement in consciousness level. The analysis exhibited substantial heterogeneity (I^2^ = 97.3%). Squares represent individual study effect sizes (MD) with 95% confidence intervals (CIs), with marker size proportional to study weight; horizontal lines indicate 95% CIs. Diamonds represent pooled estimates generated using random-effects models, with widths corresponding to 95% CIs. Statistical heterogeneity is quantified using I^2^ with associated *p*-values. (**B**) Meta-Regression Plot: Scatter plot with bubble sizes proportional to study weights examines whether intervention duration (10–28 days) predicts effect magnitude. The regression line is nearly flat, indicating no significant association between duration and GCS improvement (β = −0.010, *p* = 0.911; R^2^ = 0.0%). (**C**) Meta-Regression Plot: Scatter plot with bubble sizes proportional to study weights examines whether publication year (2007–2024) predicts effect magnitude. The regression line is nearly flat, indicating no significant association between publication year and GCS improvement (β = −0.037, *p* = 0.701; R^2^ = 0.0%). (**D**) Glasgow Outcome Scale (GOS) Forest Plot: Two studies (*n* = 213) comparing probiotics versus standard nutrition are shown [29,41,42,43,44,45,46,47,50,52]. The pooled mean difference favored probiotics (MD = 1.40, 95% CI 1.12–1.69, *p* < 0.001), indicating modest but significant improvement in functional recovery. The analysis exhibited moderate heterogeneity (I^2^ = 40.7%). Squares represent individual study effect sizes (MD) with 95% confidence intervals (CIs), with marker size proportional to study weight; horizontal lines indicate 95% CIs. Diamonds represent pooled estimates generated using random-effects models, with widths corresponding to 95% CIs. Statistical heterogeneity is quantified using I^2^ with associated *p*-values.

**Figure 5:**
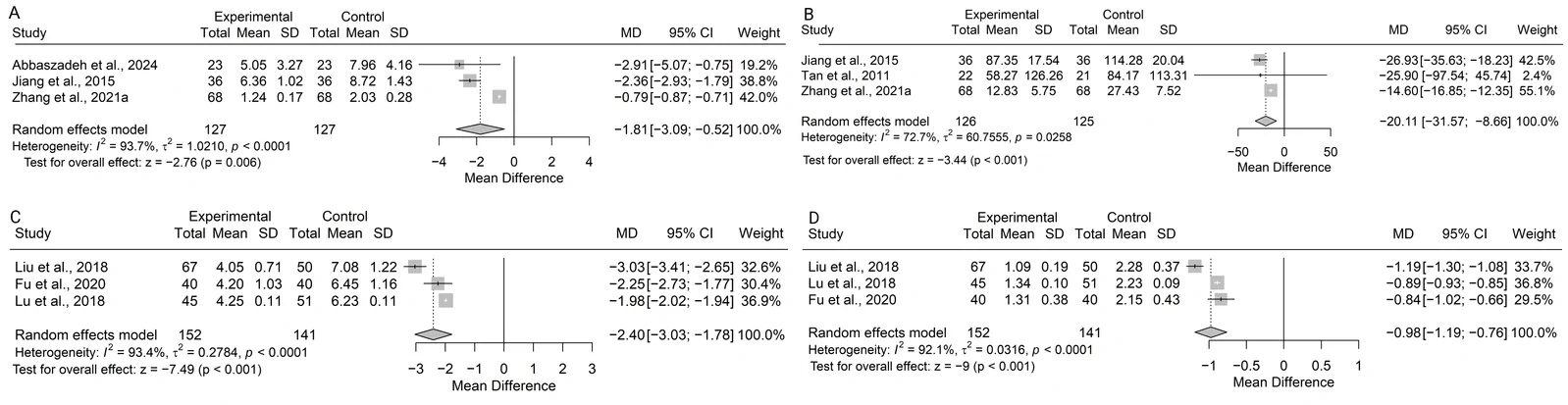
Effects of probiotics on inflammatory and gut-barrier integrity biomarkers. This figure summarizes four separate random-effects meta-analyses evaluating the impact of probiotics on key inflammatory and intestinal permeability biomarkers in patients with traumatic brain injury (TBI). (**A**) Tumor Necrosis Factor-alpha (TNF-α): Three studies (*n* = 254) demonstrated consistent reductions in circulating TNF-α following probiotic treatment in TBI patients. The pooled mean difference (MD) favored probiotics (*p* = 0.006), with significant heterogeneity (I^2^ = 93.7%). (**B**) Interleukin-6 (IL-6): Three studies (*n* = 251) showed significantly decreased IL-6 levels following probiotic treatment in TBI patients (MD = −20.11, 95% CI −31.57 to −8.66, *p* < 0.001), with heterogeneity of I^2^ = 72.7%. (**C**) Procalcitonin: In three studies (*n* = 293), probiotics markedly reduced procalcitonin (*p* < 0.001) with significant heterogeneity (I^2^ = 93.4%). (**D**) D-lactic acid: Three studies (*n* = 293) demonstrated significantly lower plasma D-lactic acid levels in probiotic-treated patients compared with controls (MD = −0.98, 95% CI −1.19 to −0.76, *p* < 0.001), although heterogeneity was high (I^2^ = 92.1%) [28,29,41,43,44,48,50,51]. Squares represent individual study effect sizes (MD) with 95% confidence intervals (CIs), with marker size proportional to study weight; horizontal lines indicate 95% CIs. Diamonds represent pooled estimates generated using random-effects models, with widths corresponding to 95% CIs. Statistical heterogeneity is quantified using I^2^ with associated *p*-values.

**Table 1:** (**A**) Study characteristics of Aim 1. (**B**) Study characteristics of Aim 2.

(**A**)							
**Author, Year**	**Age (Mean ± SD)**	**BMI (Mean ± SD)**	**Gender**	**Country**	**TBI Severity**	**Methods of Assessment**	**Journal**
Brenner et al., 2020 [39]	51.33 ±12.8	28.7 ± 6.5	TBI: 30M 4F HC: 58M 21F	USA	Moderate/severe	Region V4 16s rRNA sequencing for alpha diversity, beta diversity and relative abundance	Journal of Head Trauma Rehabilitation
Cannon et al., 2023 [19]	NA	NA	TBI: 9M 3F HC: 7–10 *	USA	Mild/moderate/severe	qPCR for relative abundance	Shock
Pyles et al., 2024 [20]	52.7 ± 7.4	28.2 ± 4.1	TBI: 5M HC: 5M	USA	Moderate/severe	qPCR array for metagenomic stability and metatranscriptomic analysis of functional profiles	Frontiers in Molecular Neuroscience
Soriano et al., 2022 [40]	19.3 ± 1.4	30.2 ± 4.8	TBI: 4M HC: 29M	USA	Mild/moderate	V1–V9 region 16s rRNA sequencing for alpha diversity, beta diversity and relative abundance	Brain, Behavior & Immunity- Health
Urban et al., 2020 [21]	33.9 ± 5.8	33 ± 4.7	TBI: 21M 1F HC: 13M 5F	USA	Moderate/severe	Region V4 16s rRNA sequencing V4 for alpha diversity, beta diversity and relative abundance	Journal of Neurotrauma
(**B**)							
**Author, Year**	**Age (Mean ± SD)**	**BMI (Mean ± SD)**	**Gender**	**Country**	**TBI Severity**	**Methods of Assessment**	**Journal**
Abbaszadeh et al., 2024 [41]	Probiotic: 33.50 ± 13.20 Control: 31.3 ± 15.96	Probiotic: 23.58 ±4.35 Control: 23.60 ± 4.04	Probiotic:16M/7F Control:14M/9F	Iran	Severe (3 to 8)	GCS; ELISA for IL-1β, IL-10, TGF-β, and TNF-a; flow cytometry for Th17 and Treg cells	Science Progress
Brenner et al., 2020 [49]	Probiotic: 37.9 Control: 36.7 ± 6.2	NA	Probiotic:16M Control:15M	United States	Mild	NSI for neurocognitive outcomes (baseline only), ELISA for biomarkers, 16s rRNA sequencing of V4 for alpha, beta, and relative abundance	Frontiers in Neurology
Fang et al., 2015 [52]	Probiotic: 40.64 ± 5.24 Control: 39.72 ± 5.01	NA	Probiotic: 24M/18F Control: 26M/16F	China	Severe (5 to 8)	GCS, and sIgA by ELISA	Chinese Journal of Microecology
Fu et al., 2020 [28]	Probiotic: 56.8 ± 11.4 Control: 55.6 ± 10.2	NA	Probiotic: 26M/14F Control: 24M/16F	China	Severe (<8)	GCS, MMSE and MoCA for cognition, Roche C702 for serum D-lactic acid and PCT	Chinese Journal of Microecology
Jiang et al., 2015 [43]	Probiotic: 42.73 ± 5.03 Control: 41.78 ± 5.12	NA	Probiotic: 21M/15F Control: 19M/17F	China	Severe (<8)	GCS; ELISA for serum IL-6 and TNF-α; D-lactic acid by enzymatic spectrophotometry; PCT by BRAHMS	Chinese Journal of Microecology
Jin et al., 2021 [42]	Probiotic: 45.66 ± 6.25 Control: 45.91 ± 5.26	NA	Probiotic: 40M/28F Control: 37M/31F	China	Severe (<8)	GCS; IgA/IgG/IgM by Biosystems A15	Progress in Modern Biomedicine
Liu et al., 2018 [44]	Probiotic: 40.6 ± 6.9, Control: 41.8 ± 7.0	NA	Probiotic 41M/39F, Control 36M/34F	China	Severe (6 to 8)	GOS, GCS, IL-6 and TNF-α by ELISA; D-lactic acid by modified enzymatic spectrophotometry; procalcitonin by quantitative immunochemiluminescence;	World Chinese Journal of Digestology
Lu et al., 2018 [50]	Probiotic: 41.29 ± 5.47 Control: 43.31 ± 6.37	NA	Probiotic: 25M/20F Control: 31M/20F	China	Severe	GOS, Th1, Th2, and Th1/Th2 by flow cytometry; intestinal barrier and inflammation by serum D-lactic acid (spectrophotometry) and PCT (immunochemiluminescence)	Chinese Journal of Immunology
Tan et al., 2011 [48]	Probiotic: 40.5 ± 13.0 Control: 40.8 ± 12.8	Probiotic: 22.3 ± 1.1 Control: 22.3 ± 1.4	Probiotic: 19M/7F Control: 21M/5F	China	Severe (5 to 8)	ELISA for serum IL-10, IL-6, and CRP	Critical Care
Xiong et al., 2013 [47]	Probiotic: 48.80 ± 8.48 Control: 49.09 ± 8.77	NA	Probiotic: 15M/2F Control: 16M/5F	China	Severe (5 to 8)	GCS and ELISA for CRP	Journal of Third Military Medical University
Yang et al., 2017 [31]	Probiotic: 38.9 ± 11.3 Control: 39.4 ± 10.5	NA	Probiotic: 24M/19F Control: 25M/18F	China	Severe	GOS, immunoturbidimetry for CD3+ and CD4+, flow cytometry for Th1/Th2	Chinese Journal of Microecology
Yu et al., 2007 [46]	Probiotic: 35.6 ± 8.3 Control: 34.6 ± 5.2	NA	Probiotic: 72M/28F Control: 74M/26F	China	Severe (<8)	GCS	Chinese Journal of Clinical Nutrition
Zhang et al., 2017 [45]	Probiotic: 49.7 ± 4.9 Control: 50.3 ± 5.1	NA	Probiotic: 23M/12F Control: 21M/14F	China	Severe (<8)	GCS, ELISA for IL-6 and IL-8	Chinese Journal of Microecology
Zhang et al., 2021 [51]	Probiotic: 46.85 ± 5.22 Control: 45.64 ± 5.31	Probiotic: 24.86 ± 3.09 Control: 25.12 ± 3.26	Probiotic: 22 M/21 F Control: 23 M/19 F	China	Severe TBI (GCS 3–8)	CD4+, CD8+, IgG, IgM, and IgA	Journal of Shandong University (Health Sciences)
Zhang et al., 2021a [29]	Probiotic: 41.54 ± 5.19 Control: 41.25 ± 5.28	NA	Probiotic: 40M/28F Control: 38M/30F	China	Severe (5 to 8)	GCS; ELISA for biomarkers; flow cytometry for levels of serum CD3+,4+,8+, and CD4+/CD8+	Progress in Nutrition

* Healthy control samples were purchased from a biobank, Discover Life Sciences. rRNA: Ribosomal RNA; qPCR: quantitative polymerase chain reaction; TBI: traumatic brain injury; BMI: body mass index; HC: healthy controls; NA: not available. ELISA: Enzyme-Linked Immunosorbent Assay; IL-1β: Interleukin-1 beta; IL-10: Interleukin-10; IL-8: Interleukin-8; TGF-β: Transforming Growth Factor-beta; TNF-α: Tumor Necrosis Factor-alpha; Th17: T helper 17 cells; Treg: Regulatory T-cells; NSI: Neurobehavioral Symptom Inventory; GCS: Glasgow Coma Scale; sIgA: Secretory Immunoglobulin A; MMSE: Mini-Mental State Examination; MoCA: Montreal Cognitive Assessment; IL-6: Interleukin-6; PCT: procalcitonin; IgA: Immunoglobulin A; IgG: Immunoglobulin G; IgM: Immunoglobulin M; CD3+: T-cells expressing CD3 protein complex on surface; CD4+: T-cells expressing CD4 protein complex on surface; CD8+: T-cells expressing CD8 protein complex on surface; CD4+/CD8+: CD4-positive to CD8-positive T-cell ratio; V4: Variable Region 4 of the 16S rRNA gene; GOS: Glasgow Outcome Scale; Th1: T helper 1 cells; Th2: T helper 2 cells; CRP: C-reactive protein.

**Table 2:** Risk of bias analysis for Aim 1.

Study	Representativeness of the Exposed Cohort	Selection of the Non-Exposed Cohort	Ascertainment of Exposure	Demonstration That Outcome of Interest Was Not Present at Start of Study	Comparability of Main Factor	Comparability of Additional Factors	Assessment of Outcome	Was Follow-Up Long Enough for Outcomes to Occur?	Adequacy of Follow-Up of Cohorts
Brenner et al. 2020 [39]	*	*	*	*	/	*	*	*	*
Cannon et al. 2023 [19]	*	*	*	*	/	*	*	/	*
Pyles et al. 2024 [20]	*	*	*	*	/	/	*	*	*
Soriano et al. 2022 [40]	*	*	*	*	/	*	*	*	*
Urban et al. 2020 [21]	*	*	*	*	/	*	*	/	*

Under each criterion, an article was awarded an ‘*’ if it met the criteria, or a ‘/’ if it fell short of the criterion for that category.

**Table 3:** (**A**) Risk of bias assessment for RCTs in Aim 2. (**B**) Risk of bias assessment for observational studies in Aim 2.

(**A**)									
**Study**	**Study Design**	**Risk of Bias Arising from the Randomization Process**	**Risk of Bias due to Deviations from the Intended Interventions (Effect of Assignment to Intervention)**	**Missing Outcome Data**	**Risk of Bias in Measurement of the Outcome**	**Risk of Bias in Selection of the Reported Result**	**Overall Risk of Bias**		
Abbaszadeh et al. 2024 [41]	RCT	Low	Low	Low	Low	Low	Low		
Brenner et al. 2020 [49]	RCT	Some concerns	Low	Some concerns	Low	Some concerns	Some concerns		
Fang et al. 2015 [52]	RCT	Some concerns	Low	Low	Some concerns	Some concerns	Some concerns		
Jiang et al. 2015 [43]	RCT	Some concerns	Low	Low	Low	Some concerns	Some concerns		
Jin et al. 2021 [42]	RCT	High	Some concerns	Low	High	Some concerns	High		
Tan et al. 2011 [48]	RCT	Low	Low	Low	Low	Low	Low		
Xiong et al. 2013 [47]	RCT	Low	Low	Low	Low	Low	Low		
Yang et al. 2017 [31]	RCT	Low	Some concerns	Low	High/some	Some concerns	Some/high		
Yu et al. 2007 [46]	RCT	Some concerns	Some concerns	Low	Low	Some concerns	Some concerns		
Zhang et al. 2017 [45]	RCT	Some concerns	Low	Low	High/some	Some concerns	Some/high		
Zhang et al. 2021a [29]	RCT	Low	Low	Low	Some concerns	Some concerns	Some concerns		
(**B**)									
**Study**	**Representativeness of the Exposed Cohort**	**Selection of the Non-Exposed Cohort**	**Ascertainment of Exposure**	**Demonstration That Outcome of Interest Was Not Present at Start of Study**	**Comparability of Main Factor**	**Comparability of Additional Factors**	**Assessment of Outcome**	**Was Follow-Up Long Enough for Outcomes to Occur?**	**Adequacy of Follow-Up of Cohorts**
Fu et al. 2020 [28]	*	*	*	*	/	*	*	/	*
Liu et al. 2018 [44]	*	*	*	*	/	*	*	*	*
Lu et al. 2018 [50]	*	*	*	*	/	*	*	*	*
Zhang et al. 2021 [51]	*	*	*	*	/	*	*	*	*

Under each criterion, an article was awarded an ‘*’ if it met the criteria, or a ‘/’ if it fell short of the criterion for that category.

## Data Availability

Data will be made available on request.

## Supplementary Materials

The following supporting information can be downloaded at: https://www.mdpi.com/article/10.3390/biology15191730/s1. Figure S1: Leave-one-out sensitivity analysis of alpha diversity. Figure S2: Sensitivity analysis of Glasgow Coma Scale by excluding high-risk randomized controlled trials. Figure S3: Forest plots of (A) IL-10, (B) CRP, (C) circulating CD3+ and (D) CD4+ T-cell levels. Figure S4: Funnel symmetry assessment of Glasgow coma scale shows symmetry, reflecting no possible small-study effects. Dashed diagonal lines represent pseudo 95% confidence bounds; the vertical line represents the pooled MD. File S1: Search Strategy: Search strategy of (A) Aim 1 and (B) Aim 2. Table S1: Excluded studies in full-text review in (A) Aim 1 and (B) Aim 2. Table S2: The table presents data for various phyla derived from a meta-analysis of phylum-level relative abundance in patients with traumatic brain injury compared to controls. Table S3: The table presents data for various genus derived from a meta-analysis of genus-level relative abundance in patients with traumatic brain injury compared to controls. Table S4: The table presents data for various species derived from a meta-analysis of species-level relative abundance in patients with traumatic brain injury compared to controls. Table S5: Certainty assessment for (A) GCS and (B) GOS. Table S6. Neurocognitive Outcome in selected studies.
