# Supplementary material for: Gut Microbiome Disruption in Traumatic Brain Injury and Therapeutic Potential of Probiotics: A Systematic Review and Meta-Analysis
Source: Biology (Basel). 2026 Oct 1;15(19):1730. doi: 10.3390/biology15191730 (PMC13663378; doi:10.3390/biology15191730)
Supplement: Supplementary file 1 [file biology-15-01730-s001.zip › Table S6.pdf]

Table S6. Neurocognitive Outcome in selected study.

Author	Sample size	Group	Metric	Mean	Sd	P-value	Notes
Fu et al., 2020 [28]	40	Pro	MMSE	24.65	2.08	<0.001	NA
Fu et al., 2020 [28]	40	Control	MMSE	22.16	1.95	<0.001	NA
Fu et al., 2020 [28]	40	Pro	MoCA	26.61	2.17	<0.001	NA
Fu et al., 2020 [28]	40	Control	MoCA	24.35	1.83	<0.001	NA
