# Supplementary material for: Gut Microbiome Disruption in Traumatic Brain Injury and Therapeutic Potential of Probiotics: A Systematic Review and Meta-Analysis
Source: Biology (Basel). 2026 Oct 1;15(19):1730. doi: 10.3390/biology15191730 (PMC13663378; doi:10.3390/biology15191730)
Supplement: Supplementary file 1 [file biology-15-01730-s001.zip › File S1 - Search strategy.pdf]

Search Strategy 1

Aim 1 – Gut Microbiome changes in TBI pateints

Database: Ovid MEDLINE(R) and Epub Ahead of Print, In-Process, In-Data-Review & Other Non-Indexed Citations, Daily and Versions <1946 to August 29, 2025>

1 exp Gastrointestinal Microbiome/ 58612

2 ((gut or gastrointestinal or intestinal or feacal or fecal or stool) adj3 (microbiome or microbiota or ecosystem or bacteria or flora or microflora or dysbiosis)).ti,ab. 120295

3 1 or 2 129097

4 Brain Injuries/ or Brain Concussion/ or Brain Hemorrhage, Traumatic/ or Brain Injury, Chronic/ or Diffuse Axonal Injury/ 71124

5 ("brain injur*" or TBI or TBIs or "Traumatic brain injury" or "hypoxic brain damage" or "diffuse axonal injur*" or "head injur*" or "brain contusion" or concussion).ti,ab. 130741

6 ((head or crani* or cerebr* or brain* or intracran* or inter-cran* or skull* or intracran* or intercran* or subarachnoid*) adj3 (injur* or trauma* or damag* or wound* or fracture* or contusion* or pressure or damag* or concussion or coma)).ti,ab. 226339

7 4 or 5 or 6 252817

8 3 and 7 582

9 (Animals/ or Models, Animal/ or Disease Models, Animal/) not Humans/ 5334351

10 ((animal or animals or canine* or dog or dogs or feline or hamster* or lamb or lambs or mice or monkey or monkeys or mouse or murine or pig or pigs or piglet* or porcine or primate* or rabbit* or rats or rat or rodent* or sheep* or veterinar*) not (human* or patient*)).ti,kf,jw. 2691937

11 9 or 10 5874361

12 8 not 11 285

Link: https://ezproxy.library.yorku.ca/login?url=http://ovidsp.ovid.com/ovidweb.cgi?T=JS&NEWS=N&PAGE=main&SHAREDSEARCHID=1TDr59dUj0FxgoXmZoWShud6W1zJ5gVhDoKuuvHNxbACsi0dbCb5qDZgJubW0Q0UY

Database: Embase <1974 to 2025 August 28>

1 exp Gastrointestinal Microbiome/ 138353

2 ((gut or gastrointestinal or intestinal or feacal or fecal or stool) adj3 (microbiome or microbiota or ecosystem or bacteria or flora or microflora or dysbiosis)).ti,ab,kw. 152264

3 1 or 2 187458

4 Brain Injuries/ or Brain Concussion/ or Brain Hemorrhage, Traumatic/ or Brain Injury, Chronic/ or Diffuse Axonal Injury/ 265470

5 ("brain injur*" or TBI or TBIs or "Traumatic brain injury" or "hypoxic brain damage" or "diffuse axonal injur*" or "head injur*" or "brain contusion" or concussion).ti,ab. 187970

6 ((head or crani* or cerebr* or brain* or intracran* or inter-cran* or skull* or intracran* or intercran* or subarachnoid*) adj3 (injur* or trauma* or damag* or wound* or fracture* or contusion* or pressure or damag* or concussion or coma)).ti,ab. 303610

7 4 or 5 or 6 503417

8 3 and 7 1066

9 (animal or animals or canine* or dog or dogs or feline or hamster* or lamb or lambs or mice or monkey or monkeys or mouse or murine or pig or pigs or piglet* or porcine or primate* or rabbit* or rats or rat or rodent* or sheep* or veterinar*).ti,kw,dq,jx. not (human* or patient*).mp. 2298323

10 (exp animal/ or exp juvenile animal/ or adult animal/ or animal cell/ or animal tissue/ or nonhuman/ or animal experiment/ or animal model/) not human/ 8009229

11 9 or 10 8072390

12 8 not 11 638

Link:
https://ezproxy.library.yorku.ca/login?url=http://ovidsp.ovid.com/ovidweb.cgi?T=JS&NEWS=N&PAGE=main&SHAREDSEARCHID=5GlF7CcnZw3ApYbPU8TG21a7WkTOhssMHy0NXmmUAaobvYFkIekwtwvTBgWYYfgmW

Database: Web of Science Core Collection

Date of search: 2025/08/30

#1 TS = ((gut OR gastrointestinal OR intestinal OR fecal OR faecal OR stool) NEAR/3 (microbiome OR microbiota OR dysbiosis OR bacteria OR flora OR microflora)) = 173,331

#2 TS = ("brain injury" OR "brain injuries" OR "traumatic brain injury" OR TBI OR TBIs OR "head injury" OR "head trauma" OR concussion OR "brain contusion" OR "diffuse axonal injury" OR "hypoxic brain damage" OR "cerebral injury" OR "skull fracture" OR "brain trauma") = 193,824

#3 TS = (animal OR animals OR canine* OR dog OR dogs OR feline OR hamster* OR lamb* OR lambs OR mice OR mouse OR murine OR monkey OR monkeys OR pig OR pigs OR piglet* OR porcine OR primate* OR rabbit* OR rats OR rat OR rodent* OR sheep* OR veterinary) = 7,000,991

#4 (#1 AND #2 AND #3) = 287

Database: Scopus

Date of search: 2025/08/30

TITLE-ABS-KEY((gut OR gastrointestinal OR intestinal OR fecal OR faecal OR stool) W/3 (microbiome OR microbiota OR dysbiosis OR bacteria OR flora OR microflora)) AND TITLE-ABS-KEY("brain injury" OR "brain injuries" OR "traumatic brain injury" OR TBI OR TBIs OR "head injury" OR "head trauma" OR concussion OR "brain contusion" OR "diffuse axonal injury" OR "hypoxic brain damage" OR "cerebral injury" OR "skull fracture" OR "brain trauma") AND TITLE-ABS-KEY(animal OR animals OR canine* OR dog OR dogs OR feline OR hamster* OR lamb* OR lambs OR mice OR mouse OR murine OR monkey OR monkeys OR pig OR pigs OR piglet* OR porcine OR primate* OR rabbit* OR rats OR rat OR rodent* OR sheep* OR veterinary)

Aim 2 – Effect of probiotic supplementation in TBI pateints

Database: Ovid MEDLINE(R) and Epub Ahead of Print, In-Process, In-Data-Review & Other Non-Indexed Citations, Daily and Versions <1946 to August 26, 2025>

1 Brain Injuries/ or Brain Concussion/ or Brain Hemorrhage, Traumatic/ or Brain Injury, Chronic/ or Diffuse Axonal Injury/ 71095

2 ("brain injur*" or TBI or TBIs or "Traumatic brain injury" or "hypoxic brain damage" or "diffuse axonal injur*" or "head injur*" or "brain contusion" or concussion).ti,ab. 130704

3 ((head or crani* or cerebr* or brain* or intracran* or inter-cran* or skull* or intracran* or intercran* or subarachnoid*) adj3 (injur* or trauma* or damag* or wound* or fracture* or contusion* or pressure or damag* or concussion or coma)).ti,ab. 226272

4 1 or 2 or 3 252748

5 Probiotics/ or Bifidobacterium bifidum/ or exp Lactobacillus/ or Streptococcus Thermophilus/ or exp Saccharomyces/ or exp Bacillus/ or exp Enterococcus/ or Escherichia/ or exp Clostridium butyricum/ 270729

6 ("probiotic*" or "bifido*" or "lactobacill*" or "streptococcus thermophilus" or saccharomyce* or bacillus or enterococcus or escherichia or boulardii or Betabacterium or Lactobacteria or "lactic acid bacteria" or casei or paracasei or rhamnosus or helveticus or acidophilus or Escherichia coli or "E.Coli" or "E. Coli" or Mutaflor or Colinfant or faecalis or "Biok+" or Lacidofil or Lactogermine or "Pb Probinul" or "BIfido Triple" or "Commensal*" or yeast or Fung*).tw. 1086043

7 Prebiotics/ or Oligosaccharides/ or Dietary Fiber/ or Gum Arabic/ or Inulin/ 61391

8 ("prebiotic*" or "dietary fiber" or "dietary fibre" or "resistant starch" or polydextrose or "gum arabic" or "hi-maize" or oligosaccharides).tw. 60104

9 Synbiotics/ 1465

10 ("synbiotic*" or familact or "probinul neutro").tw. 3206

11 5 or 6 or 7 or 8 or 9 or 10 1208808

12 4 and 11 1102

Link:
https://ezproxy.library.yorku.ca/login?url=http://ovidsp.ovid.com/ovidweb.cgi?T=JS&NEWS=N&PAGE=main&SHAREDSEARCHID=6lyB8djPYY1t2RmDTqzB4O4Jlx6UYazj9hhBV2uTufq2t1MY782wsbWwlxIwonsqE

Database: Embase <1974 to 2025 August 26>

1 Brain Injuries/ or Brain Concussion/ or Brain Hemorrhage, Traumatic/ or Brain Injury, Chronic/ or Diffuse Axonal Injury/ 265490

2 ("brain injur*" or TBI or TBIs or "Traumatic brain injury" or "hypoxic brain damage" or "diffuse axonal injur*" or "head injur*" or "brain contusion" or concussion).ti,ab. 188288

3 ((head or crani* or cerebr* or brain* or intracran* or inter-cran* or skull* or intracran* or intercran* or subarachnoid*) adj3 (injur* or trauma* or damag* or wound* or fracture* or contusion* or pressure or damag* or concussion or coma)).ti,ab. 303923

4 1 or 2 or 3 503663

5 Probiotics/ or Bifidobacterium bifidum/ or exp Lactobacillus/ or Streptococcus Thermophilus/ or exp Saccharomyces/ or exp Bacillus/ or exp Enterococcus/ or Escherichia/ or exp Clostridium butyricum/ 407045

6 ("probiotic*" or "bifido*" or "lactobacill*" or "streptococcus thermophilus" or saccharomyce* or bacillus or enterococcus or escherichia or boulardii or Betabacterium or Lactobacteria or "lactic acid bacteria" or casei or paracasei or rhamnosus or helveticus or acidophilus or Escherichia coli or "E.Coli" or "E. Coli" or Mutaflor or Colinfant or faecalis or "Biok+" or Lacidofil or Lactogermine or "Pb Probinul" or "BIfido Triple" or "Commensal*" or yeast or Fung*).tw. 1215224

7 Prebiotics/ or Oligosaccharides/ or Dietary Fiber/ or Gum Arabic/ or Inulin/ 88051

8 ("prebiotic*" or "dietary fiber" or "dietary fibre" or "resistant starch" or polydextrose or "gum arabic" or "hi-maize" or oligosaccharides).tw. 67915

9 Synbiotics/ 4100

10 ("synbiotic*" or familact or "probinul neutro").tw. 3913

11 5 or 6 or 7 or 8 or 9 or 10 1395399

12 4 and 11 3257

Link:
https://ezproxy.library.yorku.ca/login?url=http://ovidsp.ovid.com/ovidweb.cgi?T=JS&NEWS=N&PAGE=main&SHAREDSEARCHID=1Au9EXaQkfnB092LBLU6hTBGZBg3qi2pNnDWR8N14YBvSTIgUBDU0s0GLL8oLSG0y

Database: Global Health <1973 to 2025 Week 34>

1 Brain Injuries/ or Brain Concussion/ or Brain Hemorrhage, Traumatic/ or Brain Injury, Chronic/ or Diffuse Axonal Injury/ 0

2 ("brain injur*" or TBI or TBIs or "Traumatic brain injury" or "hypoxic brain damage" or "diffuse axonal injur*" or "head injur*" or "brain contusion" or concussion).ti,ab. 7339

3 ((head or crani* or cerebr* or brain* or intracran* or inter-cran* or skull* or intracran* or intercran* or subarachnoid*) adj3 (injur* or trauma* or damag* or wound* or fracture* or contusion* or pressure or damag* or concussion or coma)).ti,ab. 14983

4 1 or 2 or 3 15917

5 Probiotics/ or Bifidobacterium bifidum/ or exp Lactobacillus/ or Streptococcus Thermophilus/ or exp Saccharomyces/ or exp Bacillus/ or exp Enterococcus/ or Escherichia/ or exp Clostridium butyricum/ 216417

6 ("probiotic*" or "bifido*" or "lactobacill*" or "streptococcus thermophilus" or saccharomyce* or bacillus or enterococcus or escherichia or boulardii or Betabacterium or Lactobacteria or "lactic acid bacteria" or casei or paracasei or rhamnosus or helveticus or acidophilus or Escherichia coli or "E.Coli" or "E. Coli" or Mutaflor or Colinfant or faecalis or "Biok+" or Lacidofil or Lactogermine or "Pb Probinul" or "BIfido Triple" or "Commensal*" or yeast or Fung*).tw. 631312

7 Prebiotics/ or Oligosaccharides/ or Dietary Fiber/ or Gum Arabic/ or Inulin/ 21313

8 ("prebiotic*" or "dietary fiber" or "dietary fibre" or "resistant starch" or polydextrose or "gum arabic" or "hi-maize" or oligosaccharides).tw. 41249

9 Synbiotics/ 1637

10 ("synbiotic*" or familact or "probinul neutro").tw. 2884

11 5 or 6 or 7 or 8 or 9 or 10 659885

12 4 and 11 949

Database: Web of Science Core Collection

Date of search: 2025/08/28

#1 TS = ("brain injury" OR "brain injuries" OR "traumatic brain injury" OR TBI OR TBIs OR "head injury" OR "head trauma" OR concussion OR "brain contusion" OR "diffuse axonal injury" OR "hypoxic brain damage" OR "cerebral injury" OR "skull fracture" OR "brain trauma") = 193,793

#2 TS = (probiotic* OR prebiotic* OR synbiotic* OR "lactic acid bacteria" OR bifido* OR lactobacill* OR saccharomyces OR bacillus OR enterococcus OR escherichia OR "E. coli" OR "dietary fiber" OR "dietary fibre" OR inulin OR "resistant starch" OR polydextrose OR oligosaccharides OR "gum arabic" OR yeast) = 1,479,045

#3 (#1 AND #2) = 564

Link: https://www.webofscience.com/wos/woscc/summary/50ff2309-1de2-48fa-82ef-460f4f8164dd-01764a170f/relevance/1

Database: Scopus

Date of search: 2025/08/28

TITLE-ABS-KEY("brain injury" OR "brain injuries" OR "traumatic brain injury" OR TBI OR TBIs OR "head injury" OR "head trauma" OR concussion OR "brain contusion" OR "diffuse axonal injury" OR "hypoxic brain damage" OR "cerebral injury" OR "skull fracture" OR "brain trauma") AND TITLE-ABS-KEY(probiotic* OR prebiotic* OR synbiotic* OR "lactic acid bacteria" OR bifido* OR lactobacill* OR saccharomyces OR bacillus OR enterococcus OR escherichia OR "E. coli" OR "dietary fiber" OR "dietary fibre" OR inulin OR "resistant starch" OR polydextrose OR oligosaccharides OR "gum arabic" OR yeast) = 1,229
