# Supplementary material for: Gut Microbiome Disruption in Traumatic Brain Injury and Therapeutic Potential of Probiotics: A Systematic Review and Meta-Analysis
Source: Biology (Basel). 2026 Oct 1;15(19):1730. doi: 10.3390/biology15191730 (PMC13663378; doi:10.3390/biology15191730)
Supplement: Supplementary file 1 [file biology-15-01730-s001.zip › Table S1.pdf]

**Table S1.** Excluded studies in full-text review.

A. Aim 1

| Sl No. | References | Reasons |
|---|---|---|
| **1** | Albert V., Subramanian A., Agrawal D. Gut Microbiome Alterations in Patients with Severe Traumatic Brain Injury: A Feasibility Study. 2025; published online June 13. DOI: 10.1055/s-0045-1809656. | Irrelevant Population |
| **2** | Asztalos, Elizabeth V, Barrington, Keith, Lodha, Abhay, Tarnow-Mordi, William, Martin, Andrew. Lactoferrin infant feeding trial_Canada (LIFT_Canada): protocol for a randomized trial of adding lactoferrin to feeds of very-low-birth-weight preterm infants. 2020; published online Jan 29. DOI: 10.1186/s12887-020-1938-0. | Irrelevant article type |
| **3** | Beloborodova N., Buyakova I., Chernevskaya E. Is antibiotic treatment always necessary for chronic critical ill patients?. 2019; published online Jan 1. DOI: 10.1186/s40635-019-0254-1. | No article retrieval |
| **4** | DeLeon O., Ceccato H.D., Sidebottom A., Babtain N., Roy S., Koval J., Roth W.H., Beiser D.G., Mansour A., Goldenberg F.D., Chang E.B., Lazaridis C. MICROBIOME-MODIFIED METABOLITES PREDICT ACUTE BRAIN INJURY OUTCOME. 2021; published online June 28. DOI:10.1101/2024.06.24.599530. | Irrelevant outcome |
| **5** | Gao L., Zhang M., Zhou X. The effect of early enteral nutrition combined with anti-infection intervention on gut bacteria translocation-related infections in patients with severe traumatic brain injury. 2020; published online July 30. DOI: | Irrelevant outcome |
| **6** | He, Dongliang, Kang, Qin, Duan, Wei, Li, Guilan, He, Renli, Liu, Xiaoping, Gong, Xianghao. Investigating the Role of Gut-Derived Neurotoxin TMAO in PTSD Risk Following Traumatic Brain Injury. 2025; published online Aug 8. DOI: 10.62641/aep.v53i4.1885. | Irrelevant population<br><br>No control |
| **7** | Hoisington A.J., Stamper C.E., Kovacs E.J., Brenner L.A. 14. Alcohol use and the fecal microbiome among those seeking care in the emergency department for acute mild traumatic brain injury. 2024; published online . DOI: 10.1016/j.alcohol.2024.10.017 | Irrelevant article type |
| **8** | Hoisington A., Penzenik M., Stearns-Yoder K., Lowry C., Postolache T., Hoffmire C.A., Betz M., Stamper C.E., Forster J.E., Brenner L.A. GUT MICROBIOME AND INFLAMMATION AFTER EMERGENCY DEPARTMENT VISIT FOR MILD TRAUMATIC BRAIN INJURY 2024; published online DOI: | No article retrieval |
| **9** | Hortensius LM, van den Hooven EH, Dudink J, Tataranno ML, van Elburg RM, Benders MJNL. NutriBrain: protocol for a randomised, double-blind, controlled trial to evaluate the effects of a nutritional product on brain integrity in preterm infants. *BMC Pediatr*. 2021;21(1):132. Published 2021 Mar 17. doi:10.1186/s12887-021-02570-x | Irrelevant article type |
| **10** | Hou L., Zhao J., Yin L., Dai L., Deng H., Jiang L. Brain injury in premature infants may be related to abnormal colonization of early gut microbiome. 2024; published online. DOI: 10.1186/s12866-024-03643-4. | Irrelevant population |

| | | |
|---|---|---|
| **11** | *Jin B., Luo F.-F.* Clinical effects of Lactobacillus acidophilus combined with mosapride in treatment of gastrointestinal dysfunction after craniocerebral injury. 2021; published online. DOI: 0.11569/wcjd.v29.i19.1130 . | Irrelevant outcome |
| **12** | Liu F, Huang J, Hei G, Wu R, Liu Z. Effects of sulforaphane on cognitive function in patients with frontal brain damage: study protocol for a randomised controlled trial. *BMJ Open*. 2020;10(10):e037543. Published 2020 Oct 16. doi:10.1136/bmjopen-2020-037543 | Irrelevant article type |
| **13** | Li, Yihan & Pagulayan, Kathleen & Rau, Holly & Hendrickson, Rebecca & Schindler, Abigail. (2023). Association between gut microbiome composition and symptom self-report in trauma exposed OEF/OIF/OND Veterans. 10.1101/2023.11.01.23297931. | No Control |
| **14** | Li YI, Pagulayan K, Rau H, Hendrickson R, Schindler AG. Gut Microbial Composition Is Associated with Symptom Self-Report in Trauma-Exposed Iraq and Afghanistan Veterans. Neurotrauma Rep. 2025 Jan 8;6(1):1-12. doi: 10.1089/neur.2024.0011. PMID: 40012717; PMCID: PMC11850977. | No Control |
| **15** | Ott L, McClain CJ, Gillespie M, Young B. Cytokines and metabolic dysfunction after severe head injury. J Neurotrauma. 1994 Oct;11(5):447-72. doi: 10.1089/neu.1994.11.447. PMID: 7861440. | Irrelevant population |
| **16** | Pan, Li1; Xie, Lizheng2; Ma, Qingkai3; Liu, Baiyun4; Cheng, Hongwei2; Mao, Xiang2,4. Gut microbiome and serum metabolome as biomarkers of clinical severity and prognosis in patients with traumatic brain injury. Chinese Medical Journal 138(2):p 213-215, January 20, 2025. \| DOI: 10.1097/CM9.0000000000003310 | Irrelevant population |
| **17** | Pelland, Z., Zafar, A., Ay, A., & Belanger, K. (2024). Subconcussive head impacts sustained during American football alter gut microbiome diversity and composition. bioRxiv. DOI: 10.1101/2024.07.05.602235 | Irrelevant population |
| **18** | Pristner M, Wasinger D, Seki D, Klebermaß-Schrehof K, Berger A, Berry D, Wisgrill L, Warth B. Neuroactive metabolites and bile acids are altered in extremely premature infants with brain injury. Cell Rep Med. 2024 Apr 16;5(4):101480. doi: 10.1016/j.xcrm.2024.101480. Epub 2024 Mar 22. PMID: 38518769; PMCID: PMC11031385. | Irrelevant injury and population |
| **19** | Saji N, Murotani K, Hisada T, Tsuduki T, Sugimoto T, Kimura A, Niida S, Toba K, Sakurai T. The Association between Cerebral Small Vessel Disease and the Gut Microbiome: A Cross-Sectional Analysis. J Stroke Cerebrovasc Dis. 2021 Mar;30(3):105568. doi: 10.1016/j.jstrokecerebrovasdis.2020.105568. Epub 2021 Jan 7. PMID: 33423868. | Irrelevant population |
| **20** | Seki D, Mayer M, Hausmann B, Pjevac P, Giordano V, Goeral K, Unterasinger L, Klebermaß-Schrehof K, De Paepe K, Van de Wiele T, Spittler A, Kasprian G, Warth B, Berger A, Berry D, Wisgrill L. Aberrant gut-microbiota-immune-brain axis development in premature neonates with brain damage. Cell Host Microbe. 2021 Oct 13;29(10):1558-1572.e6. doi: 10.1016/j.chom.2021.08.004. Epub 2021 Sep 3. PMID: 34480872; PMCID: PMC8525911. | Irrelevant population |
| **21** | Seki D, Kirkegaard R, Osvatic J, Hausmann B, Séneca J, Pjevac P, Berger A, J Hall L, Wisgrill L, Berry D. Gut microbiota genome features associated with brain injury in extremely premature infants. Gut Microbes. 2024 Jan-Dec;16(1):2410479. doi: 10.1080/19490976.2024.2410479. Epub 2024 Oct 7. PMID: 39679616; PMCID: PMC11459832. | Irrelevant population |

| | | |
|---|---|---|
| **22** | Song B, Qiu Y, Wang Z, Tao Y, Wang M, Duan A, Xie M, Yin Z, Chen Z, Ma C, Wang Z. The Causal Relationship Between Gut Microbiomes, Inflammatory Mediators, and Traumatic Brain Injury in Europeans: Evidence from Genetic Correlation and Functional Mapping Annotation Analyses. Biomedicines. 2025 Mar 20;13(3):753. doi: 10.3390/biomedicines13030753. PMID: 40149729; PMCID: PMC11939942. | Irrelevant article type |
| **23** | Visser, M. K., Laman, J., Wang, Y., de Koning, M., Vakhtin, A., Mayer, A., Kremer, D., Bakker, S., van Goor, H., van der Naalt, J., Bourgonje, A., & Van Der Horn, H. (2025). POB.009: Persistently increased NOD2 activity with normal bacterial muramyl dipeptide (MDP) levels following mild traumatic brain injury. Journal of Neurotrauma, 42(13–14). https://doi.org/10.1089/neu.2025.86661.abstract | Irrelevant article type |
| **24** | Xian, Lina MDa; Xu, Xiaochen MDb; Mai, Yongmeng MDa; Guo, Tongwu MDb; Chen, Zhen MDc; Deng, Xiaoyan MDa,*. Dissecting causal relationships between gut microbiome, immune cells, and brain injury: A Mendelian randomization study. Medicine 103(38):p e39740, September 20, 2024. \| DOI: 10.1097/MD.0000000000039740 | Irrelevant population |
| **25** | You W, Lin Y, Wei A, et al. Gut Microbiome Differentiates Levels of Consciousness in Patients with Prolonged Disorders of Consciousness after Brain Injury. Journal of Neurotrauma. 2025;42(19-20):1775-1785. doi:10.1177/08977151251360256 | Irrelevant population |
| **26** | Zhanfeng N, Liang W, Jing K, Jinbo B, Yanjun C, Hechun X. Regulation of sleep disorders in patients with traumatic brain injury by intestinal flora based on the background of brain-gut axis. Front Neurosci. 2022 Oct 11;16:934822. doi: 10.3389/fnins.2022.934822. PMID: 36303945; PMCID: PMC9594989. | Irrelevant population |
| **27** | Zhu Y, Chen Z, You W, Wang Y, Tu M, Zheng P, Wen L, Yang X. A Retrospective Clinical Analysis of the Serum Bile Acid Alteration Caused by Traumatic Brain Injury. Front Neurol. 2021 Aug 25;12:624378. doi: 10.3389/fneur.2021.624378. PMID: 34512494; PMCID: PMC8424180. | Irrelevant outcome |
| **28** | Assistance Publique - Hôpitaux de Paris. (2024, May 30). Evolution of oropharyngeal and rectal microbiota after severe traumatic brain injury (BBAX) (ClinicalTrials.gov Identifier: NCT03965611). ClinicalTrials.gov. https://clinicaltrials.gov/study/NCT03965611 | Incomplete Trial |
| **29** | First Affiliated Hospital Xi'an Jiaotong University. (2022, May 10). Imaging of injury mechanism and interaction of intestinal bacteria in children with mild traumatic brain injury (ClinicalTrials.gov Identifier: NCT05090007). ClinicalTrials.gov. https://clinicaltrials.gov/study/NCT05090007 | Incomplete Trial |
| **30** | The University of Texas Medical Branch, Galveston. (2025, March 5). Brain and gut plasticity in mild TBI or post-acute COVID syndrome following growth hormone therapy (ClinicalTrials.gov Identifier: NCT03554265). ClinicalTrials.gov. https://clinicaltrials.gov/study/NCT03554265 | Incomplete Trial |
| **31** | Yu J, Meng F, He F, Chen F, Bao W, Yu Y, Zhou J, Gao J, Li J, Yao Y, Ge WP, Luo B. Metabolic Abnormalities in Patients with Chronic Disorders of Consciousness. Aging Dis. 2021 Apr 1;12(2):386-403. doi: 10.14336/AD.2020.0812. PMID: 33815872; PMCID: PMC7990357. | Irrelevant outcome |
| **32** | Turku University Hospital. (2025, April 15). Long-term recovery and microbiota-gut-brain axis disruption after traumatic brain injury (OVERCOME-TBI) (ClinicalTrials.gov Identifier: NCT06927063). ClinicalTrials.gov. https://clinicaltrials.gov/study/NCT06927063 | Incomplete Trial |

B. Aim 2

| SI No. | References | Reasons |
|---|---|---|
| **1.** | Albert V., Subramanian A., Agrawal D. Role of the Gut-Brain Axis in Severe Traumatic Brain Injury: Insights from Experimental Models and Clinical Studies. 2025; published online. DOI: 10.1055/s-0045-1807265 | Irrelevant Publication Type |
| **2.** | Curtis L., Epstein P. Nutritional treatment for acute and chronic traumatic brain injury patients. 2014; published online. https://pubmed.ncbi.nlm.nih.gov/24844176/ | Irrelevant Publication Type |
| **3.** | Dahyot-Fizelier C., Frasca D., Grégoire N., Adier C., Mimoz O., Debaene B., Couet W., Marchand S. Microdialysis study of cefotaxime cerebral distribution in patients with acute brain injury. 2013; published online. DOI: 10.1128/AAC.02570-12 | Irrelevant Study Design |
| **4.** | Falcão de Arruda I. S., de Aguilar-Nascimento J. E. Benefits of early enteral nutrition with glutamine and probiotics in brain injury patients. 2004; published online. DOI: 10.1042/CS20030251 | Irrelevant Intervention |
| **5.** | Fang Y.-M., Fang Y.-M. Gut microbiota and its role in brain injury: insights from experimental and clinical studies. 2015; published online. | Irrelevant Study Design |
| **6.** | French A. M., Merriman S. H. Nutritional status of a brain-injured population in a long-stay rehabilitation unit: a pilot study. 1999; published online. DOI: 10.1046/j.1365-277x.1999.00138.x | Irrelevant Study Design |
| **7.** | Giamarellos-Bourboulis E. J., Bengmark S., Kanellakopoulou K., Kotzampassi K. Pro- and synbiotics to control inflammation and infection in patients with multiple injuries. 2009; published online. DOI: 10.1097/TA.0b013e31819d979e | Irrelevant Population |
| **8.** | Guo Y., Xu M., Shi G., Zhang J. A new strategy of enteral nutrition intervention for ICU patients targeting intestinal flora. 2021; published online. DOI: 10.1097/MD.0000000000027763 | Irrelevant Population |
| **9.** | Habib T. A. M., Kassem A. B., Ahmed I. Effect of enteral nutrition on clinical outcomes in patients with traumatic brain injury. 2020; published online. | Irrelevant Population |
| **10.** | Jin B., Luo F.-F. Clinical effects of Lactobacillus acidophilus combined with mosapride in treatment of gastrointestinal dysfunction after craniocerebral injury. 2021; published online. DOI: 10.11569/wcjd.v29.i19.1130 | Irrelevant Intervention |

| | | |
|---|---|---|
| **11.** | Kotzampassi K., Giamarellos-Bourboulis E. J., Voudouris A., Kazamias P., Eleftheriadis E. Immunonutrition in patients undergoing major abdominal surgery: effects on infection and clinical outcome. 2006; published online. DOI: 10.1007/s00268-005-0653-1 | Irrelevant Population |
| **12.** | Li, Li, Yang, Hao, He, Tao, Zhu, Wen-yi, Liu, Jun, Fu, Hua, Wang, Jian. Tetralogy probiotics combined with galactooligosaccharides in prevention of diarrhea in patients with severe craniocerebral injury. 2020; published online. DOI: 10.16016/j.1000-5404.202003003 | Irrelevant Intervention |
| **13.** | Liu, Ming, Li, Yong-qi, Li, Kai. Effects of probiotics combined with HVHF on hemodynamics, immune inflammatory response and prognosis in elderly patients with severe head injury. 2020; published online. | Irrelevant Study Design |
| **14.** | Liu Y.-N. Effect of early dietary fiber-containing enteral nutrition on immune function and prognosis in patients with cerebral hemorrhage. 2017; published online. DOI: 10.11569/wcjd.v25.i36.3235 | Irrelevant Population |
| **15.** | Noshadi N., Sabet S. S., Sanaie S., Mahmoodpoor A., Tutunchi H., Naghshi S., Arefhosseini S. R., Ebrahimi-Mameghani M. The effects of seven-strain probiotic supplementation on cell adhesion molecules, oxidative stress and antioxidant parameters in patients with traumatic brain injury: a randomised controlled clinical trial. 2025; published online. DOI: 10.1017/S0007114524003234 | Irrelevant Outcome |
| **16.** | Pavelescu D., Mirea L., Grintescu I. Could selected probiotics have beneficial effects on clinical outcome of severe traumatic brain injury patients? 2014; published online. DOI: 10.1186/cc13662 | Irrelevant Outcome |
| **17.** | Ren, Xiu-Zan, Ma, Xu-Hui, Qiu, Jing-E, Lyu, Yi, Li, Qian-Qian . The effect of early enteral nutrition intervention on gastrointestinal tract infections in patients with severe craniocerebral injury. 2018; published online. | Irrelevant Intervention |
| **18.** | Rojas L., Giordano K. R., Ortiz J. B., Griffiths D., Lifshitz J. Probiotic treatment did not decrease the acute peripheral inflammatory response after traumatic brain injury (TBI). 2023; published online. | Irrelevant Publication Type |
| **19.** | Sabirov D. M., Khaidarova S. E. Nutritional support in the intensive care of patients with a closed head injury. 2019; published online. | Irrelevant Publication Type |

| | | |
|---|---|---|
| **20.** | Spindler-Vesel A., Bengmark S., Vovk I., Cerovic O., Kompan L. Effect of synbiotic therapy on gut integrity and clinical outcome in multiple-trauma patients: a prospective study. 2007; published online. DOI: 10.1177/0148607107031002119 | Irrelevant Population |
| **21.** | Tan, Min, Lu, Xiao-lan, Duan, Jun-wei, Peng, Hua, Zhu, Jing-ci . Effects of probiotics on blood glucose levels and clinical outcomes in patients with severe craniocerebral trauma. 2013; published online. DOI: 10.3760/cma.j.issn.2095-4352.2013.10.012 | Irrelevant Outcome |
| **22.** | Vergeer M. H., Mercier L. J., McIntosh S. J., Boucher C., Madden R. F., Shearer J., Debert C. T. Characterizing supplement use and dietary behaviors in adults with acquired brain injury. 2025; published online. DOI: 10.1080/02699052.2024.2436594 | Irrelevant Intervention |
| **23.** | Wang J., Yu K., Zeng Y. Early enteral nutrition intervention promotes multiple functional recovery in patients with traumatic intracerebral hemorrhage: a prospective randomized controlled study. 2023; published online. DOI: 10.1016/j.clineuro.2023.108010 | Irrelevant Study Design |
| **24.** | Wu A. G., Ying Z., Gomez-Pinilla F. Dietary supplementation with omega-3 fatty acids and curcumin promotes recovery after traumatic brain injury. 2007; published online. DOI: 10.1089/neu.2007.0313 | Animal Study |
| **25.** | Wu Y.-Y., Wu Y.-Y., Zhou J.-D., Zhou J.-D., Jiang Y., Jiang Y., Ying X.-H., Ying X.-H., Liu L.-Z., Liu L.-Z. Study on effects of probiotics combined with post pyloric feeding on nutritional status and ventilator-associated pneumonia in patients with severe craniocerebral injury. 2019; published online. | Irrelevant Intervention |
| **26.** | Yan X.-G. The effect of intestinal microflora alteration on postoperative recovery of patients with craniocerebral injury receiving enteral nutrition support. 2019; published online. | Irrelevant Intervention |
| **27.** | Yao Z.-N., Dong G.-J., Yu H., Zhang Q., Zuo D.-J., Zhang Y.-H. Effect of probiotic early enteral nutrition on nutritional status and inflammatory factors in patients with severe craniocerebral injury undergoing mechanical ventilation and analysis of related factors of ventilator-associated pneumonia. Progress in Modern Biomedicine. 2023; published online. DOI: 10.13241/j.cnki.pmb.2023.06.012 | Irrelevant Study Design |
| **28.** | Zhang J. Effect of probiotics-enhanced early enteral nutrition therapy on nutrition condition, immunity function and inflammatory factors with severe brain injury. Zhongguo Weishengtaxixue Zazhi / Chinese Journal of Microecology. 2017; published online. | Irrelevant Outcome |

| | | |
|---|---|---|
| **29.** | Zhang L., Fan R., Chang Y., Zhang Q. Effect of microecological enteral nutrition in 43 patients with severe craniocerebral injury. Journal of Shandong University (Health Sciences). 2021; published online. DOI: 10.6040/j.issn.1671-7554.0.2020.1702 | Irrelevant Intervention |
| **30.** | Effect of Prebiotic Fiber-Enriched (scFOS) Enteral Feeding on the Microbiome in Neurological Injury Trauma Patients (PreFEED Microbiome Trial). clinicaltrials.gov. 2017; published online. | Irrelevant study design |
| **31.** | Wan G, Wang L, Zhang G, Zhang J, Lu Y, Li J, Yi X. Effects of probiotics combined with early enteral nutrition on endothelin-1 and C-reactive protein levels and prognosis in patients with severe traumatic brain injury. J Int Med Res. 2020;48[3]:300060519888112. doi:10.1177/0300060519888112 | Retracted article |
