# Supplementary material for: Gut Microbiome Disruption in Traumatic Brain Injury and Therapeutic Potential of Probiotics: A Systematic Review and Meta-Analysis
Source: Biology (Basel). 2026 Oct 1;15(19):1730. doi: 10.3390/biology15191730 (PMC13663378; doi:10.3390/biology15191730)
Supplement: Supplementary file 1 [file biology-15-01730-s001.zip › Table S5.pdf]

Table S5A. Certainty assessment for GCS

Outcome	Risk of Bias	Inconsistency	Indirectness	Imprecision	Publication Bias	Effect	Certainty
Neurological and functional outcome following probiotic intervention in TBI patients Assessed with GCS	Not serious	Serious[a)]	Not serious	Serious[b)]	Not serious	(MD,2.25; 95% CI, 1.34 to 3.16)	Low (⨁⨁◯◯)

[a)]Downgraded one point as very high heterogeneity was present

[b)]Downgraded one point for wide CI indicating uncertain treatment effect

Table S5B. Certainty assessment for GOS

Outcome	Risk of Bias	Inconsistency	Indirectness	Imprecision	Publication Bias	Effect	Certainty
Neurological and functional outcome following probiotic intervention in TBI patients Assessed with GOS	Not serious	Not serious	Not serious	Not serious	Undetected / unable to assess (only two studies)	(MD,1.40; 95% CI, 1.12 to 1.69)	Low (⨁⨁◯◯)

[C)] Certainty started at low because the evidence was derived from two non-randomized observational studies assessed using the Newcastle–Ottawa Scale. No additional downgrading was applied for inconsistency, indirectness, or imprecision. Publication bias could not be meaningfully assessed because only two studies were available.
